# Gut Microbiota‐Derived 3‐Hydroxybutyrate Blocks GPR43‐Mediated IL6 Signaling to Ameliorate Radiation Proctopathy

**DOI:** 10.1002/advs.202306217

**Published:** 2024-05-14

**Authors:** Zhenhuang Ge, Chun Chen, Junyi Chen, Zhou Jiang, Lingming Chen, Yingqi Wei, Haiyang Chen, Lei He, Yi Zou, Xiaoxuan Long, Hongyu Zhan, Huaiming Wang, Hui Wang, Yongjun Lu

**Affiliations:** ^1^ Run Ze Laboratory for Gastrointestinal Microbiome Study, School of Life Sciences Sun Yat‐sen University Guangzhou 510275 China; ^2^ Department of Colorectal Surgery, The Sixth Affiliated Hospital Sun Yat‐sen University Guangzhou 510655 China; ^3^ School of Medical Technology Guangdong Medical University Dongguan 523808 China; ^4^ Department of Radiation Oncology, The Sixth Affiliated Hospital Sun Yat‐sen University Guangzhou 510655 China; ^5^ Affiliated Cancer Hospital & Institute of Guangzhou Medical University Guangzhou 510095 China; ^6^ Key Laboratory for Cell Homeostasis Cancer Research of Guangdong Higher Education Institutes Guangzhou 510095 China; ^7^ Guangdong Institute of Gastroenterology, Guangdong Provincial Key Laboratory of Colorectal and Pelvic Floor Diseases Supported by National Key Clinical Discipline Guangzhou 510655 China; ^8^ Shanghai General Hospital, School of Medicine Shanghai Jiao Tong University Shanghai 201620 China

**Keywords:** 3HB, akkermansia muciniphila, GPR43, IL6, radiation proctopathy, radioprotection

## Abstract

Radiation proctopathy (RP) is a common complication of radiotherapy for pelvic malignancies with high incidence. RP accompanies by microbial dysbiosis. However, how the gut microbiota affects the disease remains unclear. Here, metabolomics reveals that the fecal and serous concentrations of microbiota‐derived 3‐hydroxybutyrate (3HB) are significantly reduced in RP mice and radiotherapeutic patients. Moreover, the concentration of 3HB is negatively associated with the expression of proinflammatory IL6 that is increased along with the severity of radiation damage. 3HB treatment significantly downregulates IL6 expression and alleviates IL6‐mediated radiation damage. Irradiated cell‐fecal microbiota co‐culture experiments and in vivo assays show that such a radioprotection of 3HB is mediated by GPR43. Microbiome analysis reveals that radiation leads to a distinct bacterial community compared to untreated controls, in which *Akkermansia muciniphila* is significantly reduced in RP mice and radiotherapeutic patients and is associated with lower 3HB concentration. Gavage of *A. muciniphila* significantly increases 3HB concentration, downregulates GPR43 and IL6 expression, and ameliorates radiation damage. Collectively, these results demonstrate that the gut microbiota, including *A. muciniphila*, induce higher concentrations of 3HB to block GPR43‐mediated IL6 signaling, thereby conferring radioprotection. The findings reveal a novel implication of the gut‐immune axis in radiation pathophysiology, with potential therapeutic applications.

## Graphical abstract

Radiation reduces *A. muciniphila*‐derived 3HB concentration in the gut and serum, thereby elevating the expression of proinflammatory IL6 via GPR43‐mediated pathway, resulting in the exacerbation of tissue damage. This study demonstrate that intervention of gut dysbiosis and abnormal metabolomic network by gavage of probiotic *A. muciniphila* or 3HB ameliorates significantly post‐radiation tissue damage, which has potential therapeutic applications.

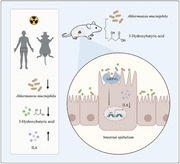

## 1 Introduction

For decades, radiotherapy has been an important component of both curative and palliative care for cancer patients, but it is also associated with serious unfavorable side effects.^[^ ^1^ ^]^ Radiation proctopathy (RP), a frequent side effect of radiation therapy for pelvic malignancies (such as tumors of the bladder, testes, prostate, rectum, cervix, and uterus) with high incidence (50–75% of patients receiving pelvic radiotherapy experience RP symptoms), is characterized by inflammation of colon tissue; however, there is no effective treatment available.^[^ ^2^ ^]^ Moreover, the fundamental mechanism behind RP development and advancement has only been partially revealed,^[^ ^2^, ^3^ ^]^ demanding additional research on RP pathogenesis, progression, and treatment. Latest studies have shown that the gut microbiota and its metabolites play a crucial role in radiation enteropathy,^[^ ^4^, ^5^, ^6^, ^7^ ^]^ however, more evidence at the molecular level is needed to provide a deeper understanding of the mechanisms, which will be instrumental to successfully develop helpful intervention strategies in patients for radiation‐induced intestinal side effects.

Although we now know that epithelial injury, the immune system, and the gut microbiota are involved in the pathogenesis of radiation enteropathy,^[^ ^8^, ^9^, ^10^, ^11^ ^]^ their interaction needs to be investigated further. Growing evidence shows that radiation leads to inflammation and gut microbiota dysbiosis. For example, tissue damage brought on by radiation is linked to higher levels of cytokine expression (e.g., interleukin (IL)1β, IL6) in both human and mouse models.^[^ ^9^, ^12^ ^]^ A large clinical study on the association of microbiota with radiation enteropathy has shown that an altered microbiota is associated with the expression of intestinal mucosal cytokines.^[^ ^4^ ^]^ These studies suggest the existence of an immunity‐microbiome axis in radiation enteropathy. As gut microbial dysbiosis, which lead to aberrant immune responses, has been associated to the development of many diseases,^[^ ^13^, ^14^ ^]^ it's conceivable that re‐establishing the balance between microbiota and host immunity is just as critical for resolving radiation enteropathy symptoms. The clinical application of microorganisms in reducing the toxicological effects of radiation seems promising, although it is still at an early stage.^[^ ^1^, ^8^ ^]^

The physiological functions of gut microbiota and metabolic systems (including host and microbial metabolism) are critical in regulating human health and diseases.^[^ ^15^, ^16^ ^]^ Metabolites produced by commensal bacteria play an important role in the host‐microbiota cross talk and affect host health.^[^ ^17^ ^]^ Growing evidence has emphasized the relevance of gut microbiota‐derived metabolites in physiology and immunological homeostasis,^[^ ^18^ ^]^ however, the characterization of metabolites and regulatory networks involved in host‐microbiota interactions in RP have not yet been elucidated.

To better understand the interactions between the radiation‐induced damage and the metabolites derived from gut microbiome, we aimed to use a mouse RP model and multi‐omics strategies to characterize the dynamics of the gut microbiota‐derived metabolites and their interplay with the host immune system, to identify and uncover the underlying mechanisms of the potential metabolites and their producing bacterium implicating in radioprotection. Our findings shed new light on the causal mechanism of the interplay between the RP damage, the gut microbiota‐derived metabolites, and host immunity, which might potentially help prevention and treatment strategies of RP in the future.

## 2 Results

### 2.1 The Concentrations of Gut Co‐Metabolite 3HB are Significantly Reduced in Feces and Serum of Irradiated Mice and Radiotherapeutic Patients

**Figure 1:**
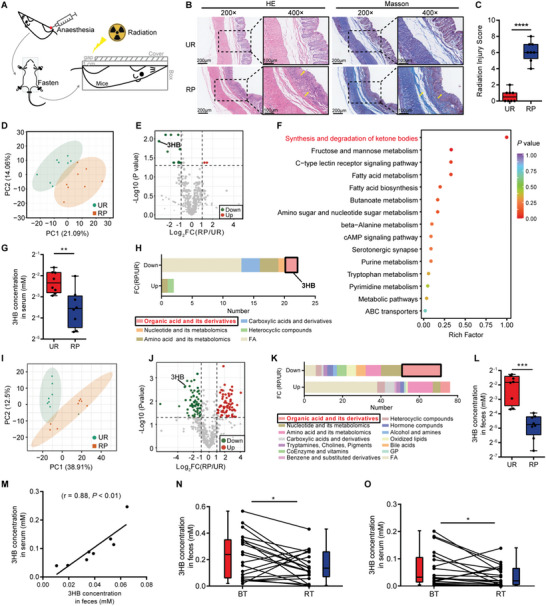
Gut co‐metabolite 3HB is downregulated in RP mice and patients. A) Experimental design schematic of localized internal rectal radiation in C57BL/6J mice. (RP, mice exposed to irradiation; UR, mice without irradiation.) B) Representative images of H&E and Masson immunostaining of the distal rectums. Insets are demonstrated in higher magnification on the right. C) Histopathological change evaluated by calculating the radiation injury score (RIS). D) PCA plot of serum metabolites from UR and RP mice. E) Volcano plot of all metabolites found in serum samples. Red points indicate metabolites with a VIP (variable importance in projection) score > 1 and an adjusted *P* < 0.05 and log2(RP/UR) > 1; green points indicate metabolites with a VIP score > 1 and an adjusted *P* < 0.05 and log2(RP/UR) < −1. F) KEGG pathway enrichment analysis of differentially enriched metabolites in serum between RP and UR mice. G) 3HB concentrations in serum between RP and UR mice. H) Cluster analysis of differentially enriched metabolites in serum. I) PCA plot of fecal metabolites from UR and RP mice. J) Volcano plot of all metabolites found in fecal samples. Red points indicate metabolites with a VIP (variable importance in projection) score > 1 and an adjusted *P* < 0.05 and log2(RP/UR) > 1; green points indicate metabolites with a VIP score > 1 and an adjusted *P* < 0.05 and log2(RP/UR) < −1. K) Cluster analysis of differentially enriched metabolites in fecal samples. L) 3HB concentrations in fecal samples between RP and UR mice. M) Correlation of 3HB between serum and fecal samples measured in RP mice. (N‐O) Detection of 3HB concentrations in fecal N) and serum O) samples derived from oncology patients before (BT) and after (RT) radiotherapy treatment. Data are representative of at least two biological replicates. Data are presented as the mean ± SEM. Animal samples were collected post 6 weeks irradiation. Patient samples were collected one day prior to radiotherapy (BT) and one day after the completion of radiotherapy (RT), respectively. N = 8 per group in mouse model. N = 20 for oncology patients. *P < 0.05, **P < 0.01, ***P < 0.001, and ****P < 0.0001 determined by the Student's *t*‐test [(C), (G), and (L)], Spearman correlation (M) and paired exact Wilcoxon test, two tailed [(N), and (O)].

To evaluate the impact of radiation treatment on the metabolite profiles and identify the metabolites related to tissue damage in RP, we took advantage of our previously developed RP mouse model using localized rectal radiation,^[^ ^19^ ^]^ which replicates the characteristics of pathological RP in humans. Radiation‐induced damage was evaluated using Hematoxylin and eosin (H&E) and Masson staining of representative lesions and radiation injury score (RIS) (**Figure** **1**A–C; Table S1, Supporting Information). We then evaluated whether such radiation damage may correlate with alternations of metabolite profiles. To address this, LC‐MS analysis of metabolites in sera isolated from unirradiated (UR) and RP mice was performed. Rarefaction analysis comparing metabolite diversity within individual subject revealed that RP mice harbor a clearly distinct metabolite profiles compared to UR mice (Figure 1D). Volcano plot analysis for all identified metabolites found 24 metabolites with significant changes (*P* < 0.05) and a confirmed variable importance (VIP score > 1) in RP mice (Figure 1E; Figure S1A; Table S2, Supporting Information). Furthermore, the differential metabolites between RP and UR mice were enriched in different metabolomic signaling pathways using KEGG enrichment analysis. Among these pathways, synthesis and degradation of ketone bodies was a top one altered in mice (Figure 1F). Importantly, the concentrations of 3‐hydroxybutyrate (3HB), a representative member in this pathway, were significant lower in serum of RP mice than UR mice (Figure 1G; Figure S1B, Supporting Information). However, no significant difference of 3HB abundance was found in liver between RP and UR mice (Figure S1C, Supporting Information), suggesting that the difference of 3HB concentration in serum is not regulated by liver. Notably, the cluster analysis of differentially expressed metabolites showed that most of the organic acids and their derivatives, including 3HB, are co‐metabolites of gut microbiota and host (Figure 1H).

We next explored whether 3HB is a co‐metabolite of gut bacteria and contribute significantly to the concentration of 3HB in the sera. To address this, mice were treated with broad‐spectrum antibiotics for removing the bacteria from the intestine (Figure S2, Supporting Information). Compared to mice drinking water only, antibiotic‐treated mice with depleted intestinal microbiomes exhibited distinct metabolite composition and reduced abundance of metabolites in feces (Figure S3A–C, Supporting Information). Interestingly, organic acid and its derivatives, especially 3HB, were significantly reduced in feces of gut microbiota deficient mice compared to control (Figure S3D,F, Supporting Information). These results reveal that 3HB is a gut co‐metabolite derived from gut microbiota. We then analyzed the metabolites in mice sera to validate whether the differences of 3HB are due to the alterations of gut microbiota composition. The results showed that gut microbiota deficient mice harbor distinct metabolite profiles compared with control mice (Figure S4A–C, Supporting Information). Remarkably, the reduced concentration of 3HB in serum was consistent with the reduction of 3HB observed in feces of gut microbiota deficient mice, and was significantly associated with the abundance of certain gut microbiota (Figure S4D–H, Supporting Information). Importantly, no significant difference of concentration of 3HB was found in the liver of gut microbiota deficient and control mice during a longitudinal follow‐up of the concentration of 3HB by location (Figure S4I; Figure S5, Supporting Information), further confirming that the altered 3HB in serum is gut derived. Altogether, these results suggest that gut microbiota‐derived 3HB is an important contributor to the concentrations of 3HB in serum.

We subsequently assessed the fecal metabolite profiles of RP mice to analyze the abundance of 3HB. PCA plots revealed that metabolite profiles in feces are also very distinct between RP and UR (Figure 1I), and 147 metabolites were identified with significant changes and VIP scores, in which 3HB was indeed significantly decreased in RP mice (Figure 1J–L; Figure S6A,B; Table S3, Supporting Information). Importantly, there was a statistically significant positive correlation between the abundance of 3HB in fecal and serum samples derived from RP mice (Figure 1M; Figure S6C; Table S4, Supporting Information). A post‐radiation longitudinal follow‐up analysis confirmed the decrease of fecal and serous 3HB concentrations (Figure S7A,B, Supporting Information). These results collectively suggested that such a reduction of 3HB in serum is caused by the decrease of 3HB in feces of RP mice. Moreover, a clinical detection of samples from patients uncovered that, compared to those before radiotherapy (BT), human subjects after radiotherapy (RT) exhibit a significantly decreased concentrations of 3HB in both fecal and serum samples (Figure 1N,O) and is not related to age and medicine used (Figure S7C–F, Supporting Information), showing an association of decreased 3HB concentration with the exposure of radiation, both in mice and human subjects.

Taken together, these data demonstrate that radiation leads to the reduction of levels of gut co‐metabolite 3HB, and suggest that 3HB may have radioprotective benefits.

### 2.2 Oral Administration of 3HB Ameliorates Radiation‐Induced Damage and Attenuates Inflammation in RP Mice

**Figure 2:**
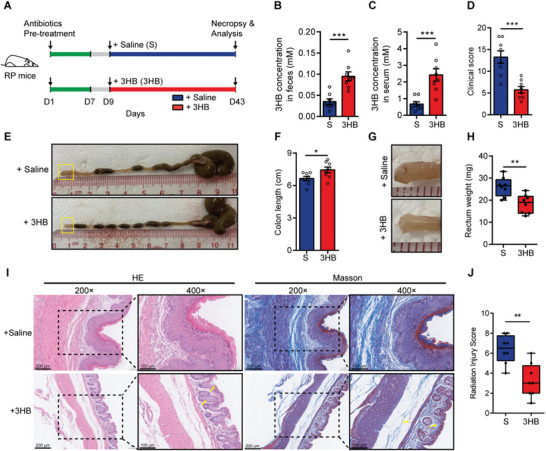
Oral administration of 3HB ameliorates radiation‐induced damage. A) Experimental diagram for determining the role of 3HB in RP mice. Construction of RP mouse model by radiation treatment. RP mice were pre‐treated with antibiotics for one week and then orally administrated with 3HB (150 mg k^−1^g body weight) or saline 3 times per week for 5 weeks. B,C) Concentrations of 3HB in fecal (B) and serum (C) samples. D) Clinical scores of the mice in each group. E,F) Representative images of the colon (E) and colon length statistics (F). Boxed regions are showed at a higher magnification in G. G,H) Representative images of the rectum (G) and rectum weight statistics (H). I) Representative images of H&E and Masson immunostaining of the distal rectum. Insets are showed at a higher magnification on the right. J) Histopathological changes evaluated by calculating the RIS score. Data are representative of at least two biological replicates. Data are presented as the mean ± SEM. N = 8 per group. Samples were collected after 5 weeks of 3HB treatment. *P < 0.05, **P < 0.01 and ***P < 0.001 determined by the Student's *t*‐test [(B), (C), (D), (F), (H), and (J)].

Subsequently, we tested whether the increased 3HB concentration can ameliorate radiation‐induced damage in RP mice. Compared to mice treated with saline, gavage of 3HB significantly increased the 3HB concentration in the feces and serum of RP mice (**Figure** **2**A–C). The effects of 3HB were assessed by histological analysis. The results showed that the clinical score (encompassing the body parameters listed in Table S5 (Supporting Information) for evaluating radiation‐induced damage, which have been proved to be proportional to disease severity^[^ ^7^ ^]^) of RP mice with 3HB treatment is significantly lower than that of those with saline treatment (Figure 2D). In addition, 3HB‐treated mice had longer colon length and decreased rectum weight. Thus, 3HB remarkably improved the pathological damage of the colon and rectum caused by radiation, inducing a protective effect against inflammation (Figure 2E–H). Furthermore, histological analysis revealed that 3HB treatment also reduces crypt damage, mucosal ulceration, immune cell infiltration, and interstitial edema (Figure 2I), which was manifested by a marked decrease in the RIS score (Figure 2J). Furthermore, 3HB treatment ameliorated radiation‐induced damage and did not induce radioprotection of the tumor in colorectal cancer (CRC) mice post‐radiation (Figure S8, Supporting Information).

Altogether, these data demonstrate that 3HB effectively ameliorates radiation‐induced damage and benefits radioprotection of mice, suggesting that 3HB is a mediator of radioprotection.

### 2.3 Reduced Concentration of 3HB is Negatively Associated with the Expression of Proinflammatory IL6 that Increased along with Disease Severity

**Figure 3:**
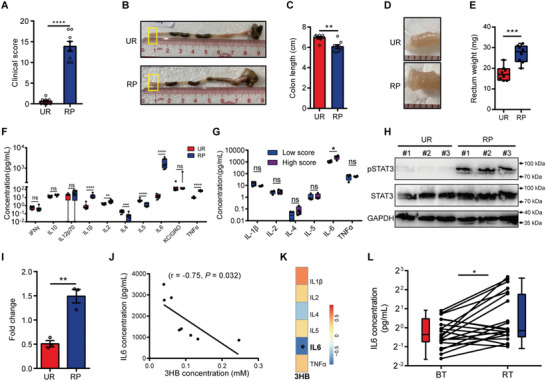
The concentration of 3HB is negatively associated with the expression of proinflammatory IL6 that increased along with the severity of radiation damage. A) Clinical score of RP and UR mice. B,C) Representative images of the colon (B) and colon length statistics (C). Boxed regions shown at higher magnification in D. D,E) Representative images of the rectum (D) and rectum weight statistics (E). F) Pooled bar graph data show the expression levels of IFNγ, IL10, IL12p70, IL1β, IL2, IL4, IL5, IL6, KC/GRO, and TNFα in the animal sera. G) The 6 differential cytokines involved in the disease severity in RP mice. H) Western blot for phosphorylated and total STAT3 in rectal tissue. Glyceraldehyde‐3‐phosphate dehydrogenase (GAPDH) served as a loading control. I) Quantitative immunoblot analysis of pSTAT3 expression (compared to STAT3 and GAPDH) calculated by ImageJ. J) Correlation between 3HB concentration and IL6 expression in sera of RP mice. K) Heatmap showing correlations between 6 differential cytokines and 3HB in RP mice. L) Detection of IL6 expression in serum samples derived from oncology patients before (BT) and after (RT) radiotherapy treatment. Data are representative of at least two biological replicates. Data are presented as the mean ± SEM. N = 8 per group in mouse model. N = 20 for oncology patients. *P < 0.05, **P < 0.01, ***P < 0.001, and ****P < 0.0001 determined by the Student's *t*‐test [(C), (E), (F), (G), and (I)], Mann–Whitney U test (A), Spearman correlation [(J), and (K)] and paired exact Wilcoxon test, two tailed (L).

Since tissue damage of RP is associated with immune system and 3HB can significantly improve the feature of colorectal inflammatory (Figure 2E–H), we then further analyzed whether RP‐induced injury may also correlate with the expression of certain cytokines. To this end, the tissues were collected from RP and UR mice to analyze the colonic and rectal tissue injury and inflammatory cytokine expression. The results showed that the clinical score proportional to disease severity is significantly higher in RP mice compared to UR mice (**Figure** **3**A). The colon length was shorter, and the rectum was thicker with increased rectal weight in RP mice, compared to UR (Figure 3B–E), indicating a severe inflammation.

Thus, we subsequently assessed the expression of 10 pro‐ and anti‐inflammatory cytokines in the sera of UR and RP mice. These cytokines were selected for analyses based on their well‐known significance in modulating inflammation response and immune system regulation.^[^ ^20^ ^]^ The expression of pro‐inflammatory cytokines IL1β, IL2, IL6, and TNFα was significantly increased in RP mice, whereas that of anti‐inflammatory cytokines IL4 and IL5 was significantly decreased (Figure 3F). However, only IL6 expression level paralleled with the severity of radiation‐induced damage (Figure 3G), suggesting its importance in response to radiation. Additionally, compared with UR mice, RP counterparts showed higher expression levels of phosphorylated (p)‐STAT3, the intracellular signaling cascade pathway activated by IL6 expression (Figure 3H,I), further confirming that the IL6 signaling pathway is activated and enhanced. We then examined whether the abundance of selected differential metabolites is correlated to abnormal inflammation status in RP mice. Interestingly, although the altered expression of six cytokines were observed in RP mice, only IL6 exhibited a stronger negative correlation to the concentration of 3HB (Figure 3J,K), suggesting its implication in mediating the radioprotection of 3HB. Importantly, the concentration of IL6 was also significantly higher in serum samples from subjects post treatment (RT) than those before radiotherapy treatment (BT) in clinical oncology patients (Figure 3L).

Collectively, these data suggest that IL6 plays a significant role in RP development, and may involve in the modulation of radioprotection of 3HB.

### 2.4 Radioprotection of 3HB is Correlated with IL6 Signaling

**Figure 4:**
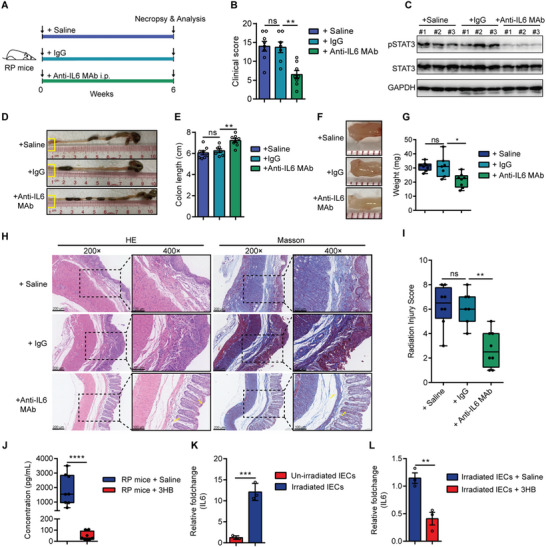
3HB can downregulate the radiation‐induced expression of IL6, a key mediator involved in RP damage. A) Experimental diagram for determining whether the blockage of IL6 signaling has protective effect in the RP mice. Construction of RP mouse model by radiation treatment. RP mice were injected with 5 mg k^−1^g anti‐IL6 MAb, or IgG antibody as control, or vehicle and injections were given every two days for 6 weeks. B) Clinical score for the mice in each group. C) Western blot for phosphorylated and total STAT3 in rectal tissue. GAPDH served as a loading control. D,E) Representative images of the colon (D) and colon length statistics (E). Boxed regions shown at higher magnification in F. F,G) Representative images of the rectum (F) and rectum weight statistics (G). H) Representative images of H&E and Masson immunostaining of the distal rectums. Insets are demonstrated in higher magnification at right. I) Histopathological change evaluated by calculating RIS score. J) Expression level of IL6 in the animal sera of the same mice as shown in Figure 2. K) Expression level of IL6 in irradiated or unirradiated HIEC‐6 epithelial cells (IECs). L) Expression level of IL6 in irradiated IECs treated with 3HB (10 mM) or saline. Data are representative of at least two biological replicates. Data are presented as the mean ± SEM. Samples were collected after 6 weeks of anti‐IL6 MAb treatment. N = 8 for saline and anti‐IL6 MAb treated groups; N = 7 for IgG treated group. *P < 0.05, **P < 0.01, ***P < 0.001, and ****P < 0.001 determined by the one‐way ANOVA with Tukey's multiple comparison test [(B), (E), (G), and (I)] and Student's *t*‐test [(J), (K), and (L)].

To investigate whether IL6 plays a causative role or a protective compensatory response in RP development, we tested whether inhibition of IL6 could ameliorated or worsen the effects of radiation‐induced damage. To this end, mice were injected with anti‐IL6 mAb or treated with IgG antibody as control (**Figure** **4**A). The results showed that RP mice treated with anti‐IL6 MAb have significantly lower clinical scores than those injected with saline or IgG (Figure 4B). Consistently, the protein abundance of pSTAT3 was also decreased in mice treated with anti‐IL6 MAb (Figure 4C), indicating that activated IL6 signaling is weakened. In addition, treatment of IL6 receptor antagonist significantly improved the colonic length and rectal weight of RP mice (Figure 4D–G), indicating a reduced inflammation symptom. Moreover, the assessment of radiation‐induced tissue damage using H&E and Masson immunostaining showed that mice receiving anti‐IL6 mAb treatment display fewer lesions and a more intact mucosa, and ameliorated RIS scores (Figure 4H,I). Thus, these data reveal that the blockade of IL6 signaling attenuates the RP damage in mice, indicating that IL6 is a major mediator of radiation‐induced RP damage.

Given the fact that blocking IL6 signaling or supplementing 3HB are both beneficial for RP mice and that 3HB has signaling roles, we next investigated whether the radioprotective effects of 3HB in RP mice is related to the inhibition of IL6 signaling. Notably, the radioprotection effects showed no significant difference between RP mice treated with 3HB alone and 3HB plus the blockade of IL6 signaling (Figure S9, Supporting Information). Indeed, 3HB treatment could significantly downregulate the expression of IL6 (Figure 4J) and the protein levels of pSTAT3 of rectal tissue in RP mice (Figure S10A,B, Supporting Information). 3HB treatment also significantly downregulated IL6 expression in irradiated HIEC‐6 epithelial cells compared to those treated with saline (Figure 4K,L). These results demonstrate that 3HB exerts radioprotection against RP damage at least in part mediated by IL6 signaling.

Collectively, these results indicate that IL6 is a major mediator of RP damage and provide evidence that the radioprotective effect of 3HB against RP damage is partially ascribed to the regulation of IL6 expression.

### 2.5 3HB Exerts Radioprotective Effect against IL6‐Mediated RP Damage via GPR43

**Figure 5:**
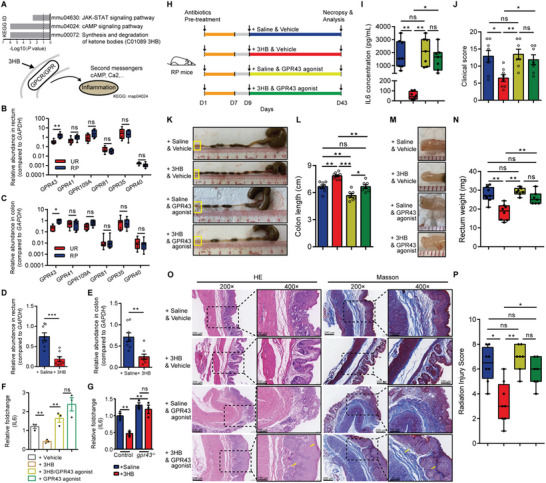
The radioprotective effect of 3HB is mediated by GPR43. A) Metabolic network integrated biochemical pathways and chemical relationships of 3HB derived from RP and UR mice. B,C) The expression of GPR receptors in the rectal (B) and colon samples (C) derived from UR and RP mice. D,E) Expression of GPR43 in the rectal (D) and colon samples (E) derived from RP mice treated with 3HB or saline. F) Expression level of IL6 in irradiated IECs treated with 3HB (10 mM) and GPR43 agonist (4CMTB, 10 µM). G) Expression level of IL6 in *gpr43^−/−^* and control cells treated with 3HB (10 mM). H) Experimental diagram for determining whether the activation of GPR43 blocks the protective effect in the RP mice. Construction of RP mouse model by radiation treatment. RP mice were pre‐treated with antibiotics before orally administrated with 3HB (150 mg k^−1^g body weight) or saline 3 times per week for 5 weeks. Mice were injected with GPR43 agonist (4CMTB, 10 mg k^−1^g body weight) or vehicle 3 times per week at the same time. I) Concentration of IL6 in the animal sera. J) Clinical score of the mice in each group. K,L) Representative images of the colon (K) and colon length statistics (L). Boxed regions are showed at higher magnification in M. M,N) Representative images of the rectum (M) and rectum weight statistics (N). O) Representative images of H&E and Masson immunostaining of the distal rectums. Insets are demonstrated in higher magnification at right. P) Histopathological change evaluated by calculating RIS score. Data are representative of at least two biological replicates. Data are presented as the mean ± SEM. Samples were collected after GPR43 agonist treatment for 5 weeks. N = 8 for the vehicle injected group; N = 7 for the GPR43 agonist injected group. *P < 0.05, **P < 0.01, and ***P < 0.001 determined by the Student's *t*‐test [(B), (C), (D), (E), and (G)], one‐way ANOVA with Tukey's multiple comparison test [(F), (I), (J), (L), (N), and (P)].

We further explored how 3HB regulates IL6 expression. We therefore performed KEGG enrichment analysis of the differentially expressed metabolites in sera and feces between RP and UR mice, respectively. The results showed that these metabolites are clustered in various pathways, in which the synthesis and degradation of ketone bodies and cAMP signaling pathways are enriched in both serum and fecal samples (**Figure** **5**A). On the other hand, as 3HB can act as a signaling molecule via G protein‐coupled receptors (GPRs) to initiate additional signaling cascades,^[^ ^21^ ^]^ including the cAMP signaling pathway (Figure 5A), we next investigated whether and which GPRs mediate the radioprotective role of 3HB. GPR43, GPR41, GPR109A, GPR81, GPR35, and GPR40 were selected for analysis based on their well‐established importance in mediating immune and metabolic functions.^[^ ^22^ ^]^ We found that, compared with UR mice, the expression of *GPR43*, but not other GPRs, in RP mice is significantly increased in colorectal tissues (Figure 5B,C). Furthermore, the expression of *GPR43* in both colonic and rectal samples from RP mice treated with 3HB was significantly reduced, compared to that in untreated controls (Figure 5D,E). These results indicate that amelioration of radiation‐induced damage by 3HB is associated with downregulated expression of GPR43.

Given that 3HB decreases the expression level of IL6, we wanted to identify the transcriptional regulator of IL6 regulated by 3HB. To address this, we initially assessed the expression of *il6* mRNA in colorectal tissue of RP and UR mice. As expected, the expression levels of IL6 were significantly higher in colorectal tissues from RP mice than those from UR mice (Figure S11A, Supporting Information). A number of known transcription factors that have been shown to regulate IL6 gene transcription were selected for analysis.^[^ ^23^ ^]^ The results showed that compared with UR mice, the expression of SP1, which is involved in regulating the chronic intestinal inflammation and apoptosis of colorectal cell lines,^[^ ^24^, ^25^ ^]^ but not other regulators, is both significantly increased in colonic and rectal tissues in RP mice (Figure S11B, Supporting Information). Indeed, RP mice treated with 3HB significantly reduced the expression of *sp1* and *il6* in both colonic and rectal samples, compared to that in untreated controls (Figure S11C,D, Supporting Information). These results indicate that 3HB inhibits radiation‐induced IL6 expression via transcriptional regulator SP1.

We subsequently tested whether GPR43 is involved in the regulation of SP1 using the irradiated cell model and co‐culture experiment. The results showed that SP1 inhibitor significantly downregulates the expression of *sp1* and *il6*, but not *gpr43* in irradiated cells (Figure S11E–G, Supporting Information). Remarkably, with or without 3HB co‐treatment, GPR43‐synthetic agonist increased the expression of *il6* and *sp1*, compared to 3HB treatment alone, in irradiated cells (Figure 5F; Figure S11H, Supporting Information). These results not only confirmed a direct role of SP1 in regulation of radiation‐induced IL6 but also indicated the control of GPR43 on SP1. Moreover, the downregulation of IL6 by 3HB was blocked by GPR43 agonist or the knockout of *gpr43* (Figure 5F,G; Figure S12, Supporting Information). Notably, these results are in consistent with those in vivo analyses in mice, indicating that the inhibition of 3HB on radiation‐induced IL6 expression is mediated by GPR43.

We next investigated whether GPR43 agonist impairs the radioprotective effect of 3HB in RP mice. RP mice pre‐treated with antibiotics were treated with or without 3HB and GPR43 agonist (Figure 5H). The results showed that, compared with those of controls, GPR43 agonist treatment significantly suppressed the effects of 3HB in downregulating the expression of IL6, reducing pSTAT3 levels, and ameliorating clinical scores (Figure 5I,J; Figure S10C,D, Supporting Information). Consistently, compared with controls, treatment of GPR43 agonist prevented the protective effects of 3HB on radiation‐induced pathological damage of the colonic and rectal tissues (Figure 5K–N). In addition, histological analysis revealed that the GPR43 agonist impairs the effects of 3HB in reducing damage of crypts, mucosal ulceration, immune cell infiltration, and interstitial edema, resulting in a high RIS score with no statistical significance compared to the controls (Figure 5O,P). Thus, blockage of the radioprotection of 3HB by activating GPR43 indicates GPR43 as a major mediator of 3HB against RP damage in mice.

Taken together, these results demonstrate that GPR43 mediates the radioprotection of 3HB by downregulating the expression of IL6 during radiation‐induced RP progression.

### 2.6 Gut Bacterium *Akkermansia muciniphila* promotes 3HB Concentration and Downregulates the Radiation‐Induced IL6 Expression

**Figure 6:**
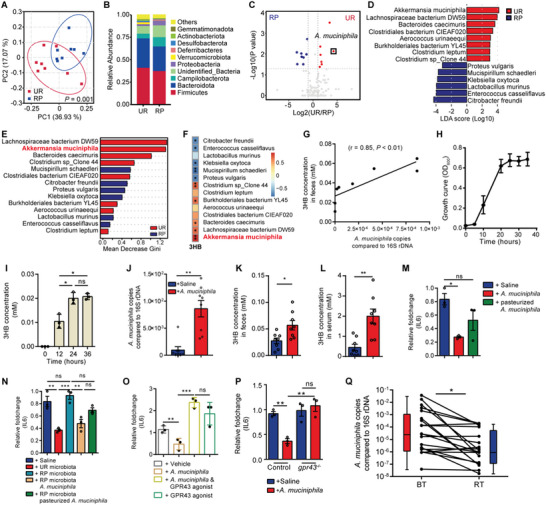
The significant reduced *A. muciniphila* in radiation‐reshaped gut bacteria is positively associated with lower 3HB concentration, can produce 3HB and downregulate IL6 expression via GPR43. A) PCoA plot (based on weighted UniFrac distances). B) The relative abundance of gut bacteria at phylum level in the fecal samples. C) Volcano plot of all bacterial species found in fecal samples. Red points indicate species with an adjusted P < 0.05 and log2(UR/RP) > 1; green points indicate species with an adjusted P < 0.05 and log2(UR/RP) < −1. *A. muciniphila* (black box) is a more abundant species enriched in UR (i.e., significantly reduced in RP mice). D) Histogram of the linear discriminant analysis (LDA) coupled with effect size measurements (LEfSe) [LDA significant threshold (log10) > ±3] identified taxonomic biomarkers at species level between UR and RP. E) Predictive importance of selected species enriched by LEfSe analysis was assessed by random forest analysis. F) Heatmap showing correlations between 3HB concentration and the abundance of LEfSe‐enriched species in RP mice. G) Correlation between 3HB concentration and *A. muciniphila* abundance in fecal samples of RP mice. H) Growth curve of *A. muciniphila* cultured in BHI under anaerobic conditions. I) Measurement of 3HB concentration in cultured *A. muciniphila* supernatant at the indicated time points. J) Analysis of *A. muciniphila* abundance in stool samples from RP mice after 5 weeks of *A. muciniphila* administration. K,L) Gavage of *A. muciniphila* increased the concentration of 3HB in fecal (K) and serum (L) samples. M) IL6 expression in irradiated IECs treated with *A. muciniphila*, inactivated *A. muciniphila* and saline, respectively. N) IL6 expression in irradiated IECs co‐cultured with fecal bacterial suspensions obtained from RP mice 6 weeks post‐radiation or from age‐matched UR mice. Viable or non‐viable *A. muciniphila* was added in the indicated group. O) IL6 expression level in irradiated IECs treated with *A. muciniphila* and GPR43 agonist (4CMTB, 10 µM). P) Expression level of IL6 in *gpr43^−/−^* and control cells treated with *A. muciniphila*. Q) Detection of *A. muciniphila* abundance in fecal samples derived from oncology patients before (BT) and after (RT) radiotherapy treatment. Data are representative of at least two biological replicates. Data are presented as the mean ± SEM. N = 8 per group in mouse model. N = 20 for oncology patients. *P < 0.05, **P < 0.01, and ***P < 0.001 determined by the Student's *t*‐test [(I), (J), (K), (L), (M), and (P)], permutation multivariate analysis of variance (PERMANOVA) test (A), one‐way ANOVA with Tukey's multiple comparison test [(N) and (O)], Spearman correlation [(F), and (G)] and paired exact Wilcoxon test, two tailed (Q).

Since gut but not liver involved in the alteration of the serous 3HB concentration in RP mice, we hypothesized that the gut microbiota may contribute to control the level of 3HB concentration. To address this, we performed 16S rDNA sequencing of fecal samples to analyze the composition of the gut bacteria in UR and RP mice. Principal co‐ordinates analysis (PCoA) revealed that RP mice harbor a distinct bacterial community relative to UR mice (**Figure** **6**A). Compared to UR, the abundance of Firmicutes and Bacteroidetes were reduced and the abundance of Proteobacteria was increased in RP mice. In particular, Verrucomicrobia, and a representative species of Verrucomicrobia, *Akkermansia muciniphila*, were significantly reduced in RP mice (Figure 6B,C). LEfSe was utilized to identify the bacterial taxa linked to radiation‐induced tissue injury. A total of 14 dominant bacteria showed a significant change in abundance in RP mice compared with UR mice (LDA score > 3, *p* < 0.05), among which *A. muciniphila* was the most significantly reduced species post radiation (Figure 6D). Additionally, random forest analysis showed that *A. muciniphila* displays a high Gini score, further confirming its role in RP development (Figure 6E). Notably, a decrease in the abundance of *A. muciniphila* in the features of radiation‐reshaped gut microbiota was consistent with that reported in previous study.^[^ ^7^ ^]^ We then evaluated the association between 3HB concentration and the significantly changed bacteria in RP mice to find dominant bacteria that can enrich 3HB concentration. We found that the reduction of 3HB concentration induced by radiation is significantly correlated with the decrease of the abundance of *A. muciniphila* in RP mice (Figure 6F,G; Table S6, Supporting Information). Meanwhile, mice with depleted intestinal microbiomes were used to find the bacteria that play dominant effects on the changes of 3HB concentration in feces under normal conditions. The results revealed significant correlations between the dominant bacteria with reduced abundance and six gut co‐metabolites with decreased concentration and uncovered that *A. muciniphila* abundance is positively correlated with 3HB concentration (Figure S13, Supporting Information). Therefore, these results collectively suggest that a radiation‐induced decrease of the abundance of *A. muciniphila* led to the reduction of 3HB concentration.

We thus measured the concentration of 3HB in the culture supernatant at different time points based on the growth curve of *A. muciniphila* to validate whether *A. muciniphila* can produce and promote the accumulation of 3HB. The results showed that the concentration of 3HB increased during the growth phases of *A. muciniphila*, with much higher concentrations during the stationary phase (Figure 6H,I). Moreover, Kyoto Encyclopedia of Genes and Genomes (KEGG) analysis and previous studies showed that FabG might be involved in the biosynthesis of 3HB,^[^ ^26^, ^27^ ^]^ and the presence of an expressible *fabG* gene in *A. muciniphila* was confirmed by PCR and qPCR analyses, respectively (Figure S14A,B, Supporting Information). When treated with epigallocatechin gallate (EGCG), an inhibitor of FabG,^[^ ^28^ ^]^ the 3HB concentration in the culture supernatant of the bacteria in stationary phase was decreased with a EGCG concentration dependent manner, whereas the survival and growth rate of *A. muciniphila* did not affect (Figure S14C,D, Supporting Information), further confirming that *A. muciniphila* can produce and accumulate 3HB. Furthermore, gavage of *A. muciniphila* significantly increased fecal and serous 3HB concentrations in the RP mice (Figure 6J–L). Thus, these data collectively indicate that gut *A. muciniphila* is an important contributor to affect 3HB levels in RP mice.

Next, we tested whether *A. muciniphila* can play a role in the regulation of radiation‐induced IL6 expression. The irradiated cells treated with live but not heat‐inactivated *A. muciniphila*, resulted in significant downregulation of the expression of IL6 (Figure 6M). IL6 level was higher in the irradiated cells treated with fecal suspensions derived from RP mice than those treated with that derived from UR mice. The fecal suspension obtained from RP mice showed no significant difference compared to the saline group, which may be due to the lack of *A. mucinipila*. Moreover, supplementation of live but not inactivated *A. muciniphila* into the microbiota derived from RP mice significantly reduced IL6 expression in co‐cultured irradiated cells (Figure 6N). We also studied whether GPR43 is involved in the downregulation of IL6 expression by *A. muciniphila* treatment. Compared with controls, *A. muciniphila* treatment significantly downregulated IL6 expression, whereas *A. muciniphila* plus the GPR43 agonist blocked the decrease of IL6 expression (Figure 6O; Figure S15, Supporting Information). Importantly, knockout of *gpr43* in HIEC‐6 cells impaired the ability of *A. muciniphila* on downregulation of radiation‐induced IL6 expression (Figure 6P). Therefore, *A. muciniphila* can directly downregulate radiation‐induced expression of IL6 via GPR43‐mediated pathway. Like the results from mice, the abundance of fecal *A. muciniphila* was significantly lower in clinical oncology patients after radiotherapy treatment (BT) than those before treatment (RT) (Figure 6Q). These results indicate that decreased *A. muciniphila* levels are related to radiation injury, and suggest that gavage of *A. mucinihila* may provide radiation protection.

Taken together, these results demonstrate that *A. muciniphila*, an important contributor of 3HB levels, can also downregulate IL6 expression through GPR43‐mediated pathway, suggesting a potential radioprotective benefits for RP.

### 2.7 Gavage of *A. muciniphila* Increases 3HB Concentration, reduces IL6 Expression and Ameliorates Radiation‐Induced Damage in RP Mice

**Figure 7:**
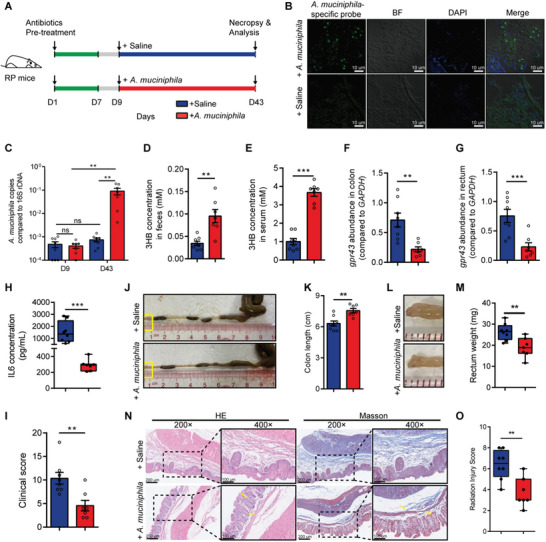
Gavage of *A. muciniphila* increases 3HB concentration, reduces IL6 expression and ameliorates radiation‐induced damage in RP mice. A) Experimental diagram for determining the role of *A. muciniphila* in RP mice. Construction of RP mouse model by radiation treatment. RP mice were pre‐treated with antibiotics for one week before orally administrated with *A. muciniphila* (2 × 10^8^) or saline 3 times per week for 5 weeks. B) Representative fluorescent in situ hybridization (FISH) and confocal microscopic imaging analysis of *A. muciniphila* (green) in the intestinal mucosa using an *A. muciniphila*‐specific probe. DAPI (4′, 6‐diamidino‐2‐phenylindole) was used for nuclear staining (blue). Scale bars, 10 µm. C) Analysis of *A. muciniphila* abundance in stool samples from mice at days 9 and 43 after antibiotic treatment. (D‐E) 3HB shows much higher concentrations in fecal D) and serum E) samples from mice with oral administration of *A. muciniphila* than that treated with saline. F,G) Expression of GPR43 was assessed by qPCR‐based analysis using mRNA extracted from the colon (F) and rectal samples (G). H) The expression of IL6 level in the animal sera. I) Clinical score of the mice in each group. J,K) Representative images of the colon (J) and colon length statistics (K). Boxed regions shown at higher magnification in L. L,M) Representative images of the rectum (L) and rectum weight statistics (M). N) Representative images of H&E and Masson immunostaining of the distal rectums. Insets are demonstrated in higher magnification at right. O) Histopathological change evaluated by calculating RIS score. Data are representative of at least two biological replicates. Data are presented as the mean ± SEM. Samples were collected after *A. muciniphila* treatment for 5 weeks. N = 8 for the saline treated group and N = 7 for the *A. muciniphila* treated group. *P < 0.05, **P < 0.01 and ***P < 0.001 determined by the Student's *t*‐test [(D), (E), (F), (G), (H), (I), (K), (M), and (O)], two‐way ANOVA with Tukey's multiple comparison test (C).

We then investigated whether *A. muciniphila* can confer radioprotective effects against RP in mice. To address this, RP mice pre‐treated with antibiotics were orally administrated *A. muciniphila* or saline (**Figure** **7**A). Fluorescence in situ hybridization (FISH) analysis by using an *A. muciniphila*‐specific probe showed that *A. muciniphila* cells colonize the intestinal epithelium of mice fed with *A. muciniphila*, compared with saline‐treated mice (Figure 7B). Colonization of *A. muciniphila* in colonic mucosa were also observed by transmission electron microscopy (Figure S16A, Supporting Information). Furthermore, the increased colonization of *A. muciniphila* was confirmed using *A. muciniphila*‐specific qPCR (Figure 7C). These results indicate that *A. muciniphila* indeed can colonize in intestinal compartment upon gavage.

Notably, gavage of *A. muciniphila* significantly increased the fecal and serous 3HB concentrations in the mice (Figure 7D,E). The expression of *GPR43* in both colonic and rectal samples from RP mice treated with *A. muciniphila* was also significantly reduced, compared to that in untreated controls (Figure 7F,G). Indeed, compared to mice fed with saline, mice fed with *A. muciniphila* resulted in downregulated expression of IL6 and pSTAT3 in rectal tissues and lower clinical scores (Figure 7H,I; Figure S16B,C, Supporting Information). As expected, *A. muciniphila* treatment significantly prevented shortening of colonic length and thickening of rectal tissue (Figure 7J–M). Moreover, pathological and histological analyses showed that gavage of *A. muciniphila* remarkably protects the distal colorectal tissue from radiation‐induced damage (Figure 7N,O). Furthermore, *A. muciniphila* treatment ameliorated radiation‐induced damage and did not induce radioprotection of the tumor in CRC mice post‐radiation (Figure S17, Supporting Information). Thus, these results reveal that *A. muciniphila* confers radioprotective effects on radiation‐induced RP damage by increasing the fecal and serous 3HB concentration, downregulating the expression of GPR43 and IL6.

## 3 Discussion

Increasing evidence has highlighted that immune system and the gut microbiota are involved in the pathogenesis of RP,^[^ ^8^, ^9^, ^10^, ^11^ ^]^ and that metabolites produced by commensal bacteria play an important role in the host‐microbiota cross‐talk. However, the relevance and characterization of regulation between gut microbiota and metabolomic involved in physiology and immunological homeostasis of RP have not yet been completely elucidated. This study demonstrated that dysbiosis and abnormal status of metabolic systems (including host and microbial metabolism) induced by radiation are related to the pathogenesis of RP, and that *A. muciniphila* and 3HB contribute substantially to radioprotection in mice, which is associated with the expression of proinflammatory cytokine IL6.

Our study showed that the metabolite profiles of RP mice is characterized by significant reduced concentration of 3HB, a gut co‐metabolite derived from commensal bacteria, higher levels of IL6, and severe tissue damage. We further provided evidence that gut microbiota‐derived 3HB plays a radioprotective role in intestinal inflammation and tissue damage. Mechanistically, gavage of 3HB significantly ameliorates radiation‐induced damage by downregulating GPR43‐mediated IL6 expression in RP mice. Moreover, our study showed that the gut microbiome pattern of RP mice is characterized by the reduction of core species, particularly *A. muciniphila* which serves as an important contributor for 3HB concentration. We confirmed that gavage of *A. muciniphila* increases 3HB concentration of mice and contributes substantially to radioprotection. Our findings contribute to advance our knowledge of the link between the RP disease mechanism and gut microbiome, and provide potential prevention and treatment for alleviating clinical radiation‐induced damage.

As an initial step of this study, we attempted to identify the metabolites and inflammatory cytokines linking to the pathogenesis of RP. Our results demonstrated that radiation can alter the fecal and serous metabolite profiles of RP mice, in which the concentration of 3HB is significantly reduced. Radiation also increases the expression of inflammatory cytokine IL6 that exhibits significant negative correlation with 3HB. Previous study^[^ ^12^ ^]^ and our results show that the expression of IL6 is correlated with the severity of radiation‐induced damage and therefore is a good marker of RP. Although 3HB has been shown to have significant therapeutic effects in colitis and colorectal cancer,^[^ ^29^, ^30^ ^]^ whether it serves as an immune effector remains unknown. We confirmed that 3HB directly downregulates radiation‐induced IL6 expression and exerts a radioprotective effect. This result is also be supported by a previous study demonstrating that treatment with 3HB suppresses the levels of inflammatory cytokines IL1β, IL6, and IL8 in human placental tissue culture.^[^ ^31^ ^]^ Another study reported that 3HB reduced NLRP3 inflammasome‐mediated IL1β and IL18 production in human monocytes.^[^ ^32^ ^]^ These findings suggest that 3HB is an immune effector and may be a promising mediator of radioprotection. In addition to 3HB, we also identified other metabolites whose levels were reduced in RP mice. Further studies are required to investigate whether these compounds have radioprotective properties.

The current data using our RP mouse model demonstrated that irradiation leads to dysbiosis, which is consistent with previous findings.^[^ ^9^, ^10^, ^11^ ^]^ Alterations in intestinal microbiota were observed in patients with RP, but a causal association with disease activity remains limited.^[^ ^1^ ^]^ Our research revealed for the first time that radiation treatment impairs colonization and abundance of *A. muciniphila*. In addition, the current study shows that the abundance of *A. muciniphila* is positively correlated with the concentration of 3HB in fecal and serous samples from RP mice, and both in vitro and in vivo experiments confirmed that *A. muciniphila* exhibits a dominant role in mediating the accumulation of 3HB. Therefore, at least one of the radioprotective pathways of *A. muciniphila* may be achieved by mediating the accumulation of 3HB. Moreover, other metabolites derived from *A. muciniphila* are known to attenuate proinflammatory cytokine responses.^[^ ^33^, ^34^ ^]^ Therefore, identifying more effective metabolites may provide a comprehensive understanding of the pleiotropic effects of *A. muciniphila* on radioprotection.

In vitro findings in the current study demonstrated that *A. muciniphila* has a direct down‐regulation effect on the expression of IL6 in epithelial cell line. Thus, *A. muciniphila* plays a protective role in intestinal inflammation and tissue damage, at least in part through the increase of 3HB concentration and the reduction of IL6 level. Previous studies have shown that lack or decrease in the abundance of *A. muciniphila* is linked to many diseases, including steatosis of the liver, inflammation, obesity, diabetes, and the response to cancer immunotherapies.^[^ ^35^ ^]^ Our data added a novel and effective application for *A. muciniphila* in RP treatment.

Our finding on the function of GPR43 has a potential clinical relevance. Previous studies have shown that GPR43 is extensively expressed in immune tissues and cells,^[^ ^36^ ^]^ suggesting its important role in immune responses. However, the role of GPR43 in the inflammatory response remains elusive. GPR43 may have both pro‐ and anti‐inflammatory effects, depending on the disease model employed.^[^ ^37^, ^38^, ^39^, ^40^, ^41^ ^]^ In addition, GPR43 may mediate interactions between the human host and gut microbiome.^[^ ^42^ ^]^ Our results demonstrated that activation of GPR43 blocks the downregulation of IL6 by *A. muciniphila* or *A. muciniphila*‐mediated 3HB, thus prevents the radioprotection. This result further points to the unique association of GPR43 with gut microbiota and radiation‐induced IL6 expression in the pathogenesis of RP. The relationship between GPR43 and IL6 has also been reported in other studies. For example, knockout of GPR43 reduces IL6/IL1β/TNFα levels in cells transfected with bacterial pathogen *Klebsiella pneumoniae*.^[^ ^43^ ^]^ Another study found that GPR43 activation enhances psoriasis‐like inflammation by upregulating IL6 signaling in the epidermis.^[^ ^44^ ^]^ These findings suggest that GPR43 may also play a crucial role in radiation‐induced intestinal injury. Our results will be particularly relevant for the clinical application of GPR43 agonists or antagonists in patients with RP. Therefore, future clinical trials are needed to evaluate the inhibition effect of GPR43 signaling in patients with RP.

Gut microbiota may be useful for the prevention and treatment of RP. We showed that RP mice has depletion in the Firmicutes and Bacteroidetes phyla and an increase in Proteobacteria, similar to what was observed in inflammatory bowel disease (IBD).^[^ ^45^ ^]^ Thus, dysbiosis is a common feature in IBD and RP. Moreover, we found that RP and IBD share similarities, as both are accompanied by fibrosis, inflammation, epithelial barrier breaches, and mucosal immune cell infiltration. Modulation of IBD activity by manipulating the microbiome is now gaining a lot of attention, thus it seems sense for RP to follow suit. Probiotics, and fecal microbial transplants (FMT) have all been recommended for the treatment of IBD and may also be effective for the management of RP. Attempts at bacteriotherapy in the setting of RP appear to be safe, according to several research conducted on both rodents and humans.^[^ ^46^, ^47^ ^]^

In conclusion, the results of this study demonstrate radiation‐induced changes in the microbiome and metabolome and how such an alteration impacts RP‐induced inflammation and RP progression. These findings suggest the potential of *A. muciniphila* and 3HB in the treatment of RP, and that effective treatment of RP could be achieved by modulating the gut microbiota and its interaction with the immune system. Collectively, given the fact that current therapeutic methods for remission of the adverse side effects of RP are limited and expensive, our study provides a promising clinical option for the management of the damage associated with RP.

## 4 Experimental Section

### Mice

Female C57BL/6J mice aged 6–8 weeks or C57BL/6‐APC^min/+^ mice aged 8–10 weeks were kept in a specified pathogen‐free (SPF) environment with unrestricted access to drinking water and food under temperature‐controlled settings and a 12‐hour light/dark cycle. The littermates were used in the experiments. All animal research was approved by the Institutional Animal Care and Use Committee of the Sixth Affiliated Hospital, Sun Yat‐sen University (IACUC‐20200813).

### RP Mouse Model

The mice were treated with rectal radiation using an RS2000 device (Rad Source, USA) as we previously described.^[^ ^19^ ^]^ The mice were subjected to irradiation (25 Gy; 2.14 Gy min^−1^) while being protected by a 4 mm thick lead shelter that exposed the lower pelvic area (1 cm^2^) including the rectum in the center of the field. After irradiation, the mice were kept in an SPF setting with regular feed and water. Unless otherwise specified, mice were examined for changes in body weight and other bodily parameters 6 weeks following radiation. Clinical scores were calculated based on weight reduction, physical appearance, posture, mobility, anal hair, and hydration using a minor modified cumulative scoring method (Table S5, Supporting Information).^[^ ^7^ ^]^

### Clinical Oncology Patients

Human fecal and serum samples were obtained from 20 patients with rectal cancer. All patients included in this study received the same standardized radiation therapy regimen, which was PTV‐GTV 50 Gy/25F and PTV‐CTV 45 Gy/25F for 5 weeks. The detailed information and the radiotherapy regimen of the oncology patients was also showed in Table S7 (Supporting Information). Patients with typical clinical symptoms of RP after 5‐weeks radiotherapy were enrolled in the study, which was confirmed by endoscopic examinations of patients before radiotherapy and after the completion of radiotherapy. Representative endoscopy examination showed subsequent changes of the intestinal mucosa after pelvic radiotherapy, reflecting a clear phenotype of the disease (Figure S18A, centre). After exposure and radiation, mucosal edema appeared in the rectum and colon, with discoloration and blackening of the mucosa, disappearance of normal blood vessel distribution, pallor of the mucosa, disappearance of mucosal folds, and irregular superficial ulcers. The same standard was used to collect samples from all RP patients, namely, all samples were collected one day prior to radiotherapy (BT) and one day after the completion of 5‐weeks radiotherapy (RT), respectively. Fecal samples were collected in stool collection containers and transferred immediately to a refrigerator with a temperature of −80 °C until use. After collection, blood samples were placed in a refrigerator at 4 °C, centrifuged at 3000 rpm at 4 °C for 10 minutes, and the upper serum layer was removed and stored in a −80 °C refrigerator until use. The experiment was approved by the internal review and the ethics boards of Ethics Committee of the Sixth Affiliated Hospital of Sun Yat‐sen University (2021ZSLYEC‐262) with patient informed consent.

### Sample Collection

Fecal and serum samples were collected at 6 weeks post‐radiation because the clinical scores of RP mice at 6 and 8 weeks showed no difference (Figure S18B, Supporting Information). Samples from each animal were either preserved at −80 °C for microbiota composition analyses, metabolite profiles, or processed for subsequent investigations. To do this, the samples were combined by vortex, homogenized in saline, then centrifuged at 800 rpm for 5 minutes to pellet bacterial cells. Pellets were washed in PBS, reconstituted in an equivalent volume of brain‐heart infusion (BHI) liquid media (with 50% glycerol), and kept at −80 °C for co‐culture investigations. Mice in the radiation, control, and treatment groups were put to death six weeks after being exposed to radiation. Blood was collected, centrifuged for 10 min at 3000 rpm after being held at ambient temperature for 30 min. The supernatant serum was kept at −80 °C for metabolite profiles and cytokine assays. Colonic segments and liver tissue were promptly frozen in liquid nitrogen and kept at −80 °C. The rectal tissue 1 cm above the anus was excised, washed, and sliced into two equal length specimens along the craniocaudal axis. For western blotting or quantitative PCR, one specimen was preserved in liquid nitrogen; for histopathological investigation, the second specimen was fixed in 4% formaldehyde.

### Histopathological Analysis

Rectal samples, fixed in 4% formaldehyde, were used for hematoxylin and eosin (HE) and Masson's trichrome staining. Sections of the tissues that were four microns thick were cut using a rotary microtome (Leica, Germany), placed on slides for staining and then examined under a light microscope in accordance with established techniques (DM2500, Leica). A certified pathologist double‐blindly carried out histological examinations. Collagen, cell nuclei, and cytoplasm received blue, dark purple, and red/pink, respectively, colors in the Masson's trichrome staining. After microscopic analysis of the stained slides, the radiation injury score (RIS), which was modified from Langberg et al.,^[^ ^48^ ^]^ was computed to assess the histological alterations. Such changes were based on Masson and HE staining, which revealed typical histological characteristics of RP lesions, including mucosal ulceration, infiltrating inflammatory cells, edema, vessel stenosis, and submucosal fibrosis (Table S1, Supporting Information).

### Cytokines Assay

The circulating concentrations of IFNγ, IL10, IL12p70, IL1β, IL2, IL4, IL5, IL6, KC/GRO, and TNFα were measured using the MSD V‐Plex Proinflammatory Panel 1 Mouse Kit (Meso Scale Diagnostics, Cat# K15048D). Serum cytokine IL6 level was measured using an ELISA kit (Cloud‐Clone Corp, SEA079Mu) as directed by the manufacturer's instructions.

### Fecal 16S rDNA Gene Sequencing

Fecal sample preparation and 16S rDNA gene sequencing were carried out as it was previously described.^[^ ^33^, ^34^ ^]^ The QIAamp Fast DNA stool Mini Kit (Qiagen, Cat# 51604) was used to extract bacterial DNA from fecal samples in accordance with the manufacturer's instructions. By utilizing barcoded primer pairs that targeted the V3‐V4 region of the 16S rDNA gene, fecal DNA samples were amplified by polymerase chain reaction (PCR). The Illumina NovaSeq6000 was used to sequence the PCR amplicons (Novogene Co., Ltd., China) based on standard protocols. QIIME (version 1.9.1) was used to examine the resultant bacterial sequence fragments. The 16S rDNA variable region primers used to target the region's V3‐V4 were listed in Table S8 (Supporting Information). The raw sequences were saved in the Sequence Read Archive database.

Principal co‐ordinates analysis (PCoA) plots were used to show the generated matrices. Heatmaps among gut microbiota, cytokines and metabolite results were created with the statistical computing environment R's “heatmap” function. The effect size (LEfSe) of linear discriminant analysis (LDA) was utilized to find changes in relative abundance. The Spearman's correlation coefficient was used to calculate correlations. Random forest analysis was performed using the R Studio (v 3.5.0).

### Immunoblot Analysis

Immunoblotting was carried out as it was previously reported.^[^ ^33^ ^]^ Briefly, rectal tissues collected from the indicated mice were lysed, and total protein was isolated and measured. Extracted proteins were electrophoresed in 10% SDS‐PAGE with loading buffer before being transferred to PVDF membranes (Bio‐Rad) and then put in 5% fat‐free milk to block for one hour. The membranes were treated with primary antibodies overnight at 4 °C before being incubated for 1 hour at room temperature with a secondary antibody (Cell Signaling). The protein bands were visualized using an ECL kit (Millipore). Images were captured using a Tanon5200 machine (Tanon, China). ImageJ 1.43 software was used to analyze the data.

### Antibodies

Rabbit‐signal transducer and activator of transcription 3 (STAT3) (#4904, Cell Signaling), rabbit‐phosphorylated p‐STAT3 (#9145, Cell Signaling), rabbit‐glyceraldehyde‐3‐phosphate dehydrogenase (GAPDH) (#5174, Cell Signaling), HRP‐linked anti‐rabbit IgG antibody (#7074, Cell Signaling), anti‐IL6 MAb Tocilizumab (HY‐P9917, MCE), and isotype control IgG (HY‐P70251, MCE) were used.

### IL6 Receptor Antagonist Treatment

After irradiation of RP mice, the animals were injected with 5 mg k^−1^g anti‐IL6 MAb, IgG antibody control, or saline every two days per week for six weeks. The clinical score plots and samples were then collected.

### Co‐Culture of Epithelial Cells with Microbiota

Human HIEC‐6 intestinal epithelial cell line was obtained from the American Type Culture Collection and cultured in Dulbecco's modified Eagle's medium (11965092, Gibco) with 10% fetal bovine serum (12483020, Gibco) at 37 °C in 5% CO_2_ and 95% air for 24 h. The HIEC‐6 cells were irradiated (8 Gy) using an RS2000 device. For the co‐culture experiments, the epithelial cells were seeded (5×10^5^ cells) into Nunc EasYFlask (25 cm^2^) (Thermo Scientific), and then the medium was removed and the bacterial cell suspensions (MOI = 10^2^) obtained from irradiated and un‐irradiated mice were added. For irradiated cells treated with *A. muciniphila*, active or pasteurized *A. muciniphila* (MOI = 10^2^) were added and co‐culture at 37°C in 5% CO_2_ and 95% air for 24 h. For irradiated cells co‐cultured with fecal suspensions derived from RP mice, the feces were collected from RP mice post radiation for 6 weeks as described in sample collection section, and the bacterial cell suspension were prepared and added to irradiated cells at an MOI = 10^2^, then were kept at 37°C in 5% CO_2_ and 95% air for 24 h. For irradiated cells treated with bacterial cell suspension plus *A. muciniphila* at an MOI = 10^2^, in which *A. muciniphila* was at a ratio of 2%, then were co‐cultured at 37°C in 5% CO_2_ and 95% air for 24 h. Cell samples were then collected after the co‐culture treatments.

### RNA Extraction and Real‐Time PCR

TRIzol reagent (Takara, Japan) was used to extract total RNA from tissues or cells, and a Nanodrop spectrometer was used to measure total RNA concentration. Using a Promega cDNA Synthesis Kit, first‐strand cDNA was created by following the manufacturer's instructions. To measure the expression of IL6 and GPR43, quantitative real‐time PCR was carried out in triplicate using SYBR Green. The endogenous control GADPH was utilized to standardize gene expression. The primers used for IL6, GPR43, and GAPDH were listed in Table S8 (Supporting Information). The data were evaluated using an ABI StepOnePlus real‐time PCR system (Applied Biosystems).

### Liquid culture of *A. muciniphila*

*A. muciniphila* (ATCC BAA‐835) was cultured in BHI medium at 37 °C under anaerobic conditions, as it was previously described.^[^ ^34^ ^]^ The growth curve (OD_600_) of *A. muciniphila* was established every 12 h using a Genesys spectrophotometer (Thermo Scientific). For detecting the effects of FabG on 3HB concentration, *A. muciniphila* cells in stationary phase were given escalating doses of epigallocatechin gallate (EGCG) for 24 h as previously reported.^[^ ^28^ ^]^ For gut colonization of mice by *A. muciniphila*, cultures were cleaned and concentrated in anaerobic saline containing 25% (v/v) glycerol under anaerobic conditions. In addition, an equivalent amount of *A. muciniphila* cultured in the same medium was heat‐inactivated at 70 °C for 30 minutes.

### Antibiotics Treatment

For the germ‐free mice model, a mixture of ampicillin (1 mg mL^−1^, Sigma, CAS# 7177–48‐2), neomycin sulfate (1 mg mL^−1^, Sigma, CAS# 1405–10‐3), vancomycin (0.5 mg mL^−1^, Sigma, CAS# 1404–90‐6), and metronidazole (0.2 mg mL^−1^, Sigma, CAS# 443–48‐1) was added to sanitize the mice's drinking water. Three times every week, the solutions and bottles were replaced. UR mice were drinked with antibiotics mixture or water for 6 weeks. RP mice were drinked with antibiotics mixture for 1 week after radiation, and then treated with *A. muciniphila* or 3HB for another 5 weeks. *A. muciniphila* treated group were drinked with normal water and 3HB treated group were drinked with antibiotic water in the duration of treatment. Fecal pellets were cultivated on blood agar plates, resuspended in BHI + 50% glycerol (0.1 g mL^−1^), and incubated for 48 hours at 37 °C in both aerobic and anaerobic conditions to test antibiotic sensitivity.

### A. *muciniphila* Oral Administration

For the treatment of mice using *A. muciniphila*, the cells were grown in BHI broth medium at 37 °C under anaerobic conditions, as it was previously reported.^[^ ^33^, ^34^ ^]^ Gut colonization of antibiotics pre‐treated RP mice by *A. muciniphila* was conducted by oral gavage with 200 µL bacterial suspension containing 2 × 10^8^ cells or saline 3 times per week. Clinical score plots and samples were collected after five weeks of *A. muciniphila* treatment.

### Detection of A. muciniphila

According to the previous investigation, the abundance of *A. muciniphila* in fecal samples was evaluated using quantitative PCR (qPCR).^[^ ^33^ ^]^ Following the manufacturer's instructions, genomic DNA was extracted from tissues or feces using the QIAamp DNA Tissue or Stools Mini Kit (Qiagen). SYBR Green‐based targeted qPCR devices were used. Primers were utilized as previously described.

### Fluorescence In Situ Hybridization (FISH)

FISH and immunofluorescence of *A. muciniphila* were performed as it was described previously.^[^ ^33^ ^]^ Briefly, deparaffinization was performed on paraformaldehyde‐fixed paraffin‐embedded colon tissue slices (5 mm). To identify bacterial colonization, a fluorescein‐labeled oligonucleotide probe targeting one area of the *A. muciniphila* 16S rDNA gene was utilized. As a negative control, nonspecific hybridization was detected using the non‐EUB probe. In situ hybridization was carried out at 50 °C overnight. Slides were coated with ProLong Gold with DAPI (Invitrogen), sealed with coverslips, and permitted to dry overnight at 4°C in the dark before being photographed using a confocal microscope (LSM 880 with Airyscan).

### Transmission Electron Microscopy (TEM)

Transmission electron microscopy (TEM) was used to investigate *A. muciniphila* colonization as it was described previously.^[^ ^33^ ^]^ Briefly, the intestinal tissues of mono‐colonized mice were cut into 2–3 mm cubes and promptly fixed at 4°C overnight using a 0.1 M sodium cacodylate buffer containing 3% glutaraldehyde. The samples were then implanted in epoxy resin after being post‐fixed in a 2% osmium tetroxide buffered solution. Then, the samples were processed in the manner previously described. FEI Tecnai G2 Spirit BioTwin 634 was used to create electron micrographs.

### 3HB Treatment and Determination

RP mice pretreated with antibiotics were orally gavaged with 200 µL 3HB (150 mg k^−1^g body weight) or saline 3 times per week. Clinical score plots and samples were collected after five weeks of 3HB treatment. Irradiated HIEC‐6 cells, with or without 3HB (10 mM), were cultured in 5% CO_2_ and 95% air conditions for 24 h, and then the expression of IL6 was assessed. Each gram of feces and liver tissue were homogenized with 1 mL saline. Ten minutes of centrifugation at 4 °C of 12,000 rpm to obtain the supernatants for further analysis. The concentration of 3HB in mice sera, feces and liver and supernatant of *A. muciniphila* was quantified using an ELISA kit (Cloud‐Clone Corp, CEB022Ge), as directed by the manufacturer's instructions.

### Metabolomics Analysis

The samples stored at −80°C were thawed on ice. To precipitate the proteins, 300 µL of methanol were added to 100 µL of each sample, vortexed for 3 min, and remained for 10 min at room temperature. Following 20 minutes of centrifugation at 4°C of 12,000 rpm, the supernatants were analyzed by LC‐MS analysis, following the manufacturer's instructions (Metware Biotechnology Co., Ltd. Wuhan).

The first‐ and second‐order spectra obtained by mass spectrometry were qualitatively analyzed using the self‐database, metware database (MWDB), and the public database of metabolite information. Multiple reaction monitoring (MRM) triple quadrupole mass spectrometry was used to quantify the metabolites. The statistical function prcomp inside R was used to perform unsupervised principal component analysis (PCA). VIP ≥ 1, *p*‐value < 0.05, and absolute Log2FC ≥ 1 were used to identify significantly regulated metabolites across groups. The Mann‐Whitney U‐test was used for each metabolite to compute the p‐value for significance. VIP values were calculated using the R package MetaboAnalystR and retrieved from the OPLS‐DA data, which included included score plots and permutation plots. The cor function in R was used to generate the Spearman correlation coefficient between samples, which was then shown as heatmaps. The KEGG database (http://www.kegg.jp/kegg) was used to annotate the identified metabolites, and the annotated metabolites were then linked to the KEGG pathway database. A hypergeometric *p*‐value test was used to find pathways that had significantly modulated metabolites.

### GPR43 Agonist Treatment

For irradiated HIEC‐6 cells co‐cultured with *A. muciniphila* or treated with 3HB, vehicle [10% DMSO (D2650, Sigma‐Aldrich) + 40% PEG300 (HY‐Y0873, MCE) + 5% Tween 80 (HY‐Y1891, MCE) + 45% saline] was used to dissolve GPR43 agonist to prepare 10 mM storage concentration. Irradiated cells were treated with 10 µM GPR43 agonist (4‐CMTB,^[^ ^49^ ^]^ HY‐P1125, MCE) or vehicle and then the expression of IL6 was assessed. To determine the effect of the GPR43 agonist on mice, RP mice pretreated with antibiotics were orally gavaged with 200 µL 3HB (150 mg k^−1^g body weight) or saline and were injected with 200 µL 4CMTB (10 mg k^−1^g body weight) or vehicle. Clinical scores and samples were obtained following five weeks of treatment.

### Generation of GPR43 KO in Human HIEC6 Cell Line

The CRISPR–Cas9‐based KO strategy was used for GPR43 knock out in human HIEC6 cells. The sgRNAs targeting *GPR34* were designed using Benchling. CRISPR‐Cas9 sgRNA sequences were listed in Table S8 (Supporting Information). CRISPR/Cas9 was performed using LentiCRISPRv2 vector. sgRNAs were cloned into lentiCRISPRv2 according to the lentiCRISPRv2 cloning protocol. Lentiviruses were then generated by transfection of HEK293T cells with lentiCRISPRv2‐sgGPR34 and the packaging plasmids psPAX2 and pMD2.G using Lipofectamine 3000 according to the manufacturer's instructions. Viral particles in the cell culture supernatant were filtered with 0.45 um filters to remove cellular debris. HIEC6 cells were transduced with lentiviruses in the presence of polybrene (10 ug mL^−1^), 72 h after infection, cells were selected under 2 ug mL^−1^ puromycin for 7 days. The cells were cultured for at least five passages to establish the stable cell lines.

### Data Analysis

GraphPad Prism 6 (GraphPad Software, USA) was used for statistical analysis. At least two biological replicates were conducted in vivo animal experiments, and at least three biological replicates were conducted in vitro cell, bacterial, and molecular experiments. The figure legends indicated the number of animals (n) used in the studies. One dot or lane indicated one mouse or sample. All values were shown as the mean ± SEM, with *P < 0.05, **P < 0.01, ***P < 0.001, ****P < 0.0001; ns, not significant. The D'Agostino‐Pearson omnibus test was used to determine data normal distributions. If statistical significance between two groups was not mentioned in the figure legends, it was calculated using an unpaired, two‐tailed Student's *t* test, Mann‐Whitney test, or permutation multivariate analysis of variance (PERMANOVA) test, and significance of more than two groups was established using one‐way ANOVA or two‐way ANOVA in GraphPad Prism with default setting depending on experience.

## Conflict of Statement

The authors declare no conflict of interest.

## Author Contributions

Z.G., H.M.W., H.W., Y.L. planned the experiments. Z.G., C.C., J.C., Z.J., L.C., Y.W., H.C., L.H., Y.Z., X.L., H.Z. performed the experiments and analysis. Z.G., Y.L. wrote the draft of the manuscript. All authors contributed to the edits of the manuscript.

## Supporting information

Supporting Information

## Data Availability

The data that support the findings of this study are openly available in 16S rDNA sequencing of fecal samples in radiation proctopathy mice at https://dataview.ncbi.nlm.nih.gov/, reference number 881491.
