# Supplementary material for: Gut Microbiota‐Derived 3‐Hydroxybutyrate Blocks GPR43‐Mediated IL6 Signaling to Ameliorate Radiation Proctopathy
Source: Adv Sci (Weinh). 2024 May 14;11(28):2306217. doi: 10.1002/advs.202306217 (PMC11267371; doi:10.1002/advs.202306217)
Supplement: Supplementary file 1 — Supporting Information [file ADVS-11-2306217-s001.pdf]

ADVANCED SCIENCE
Open Access

Supporting Information

for *Adv. Sci.*, DOI 10.1002/advs.202306217

Gut Microbiota-Derived 3-Hydroxybutyrate Blocks GPR43-Mediated IL6 Signaling to Ameliorate Radiation Proctopathy

Zhenhuang Ge, Chun Chen, Junyi Chen, Zhou Jiang, Lingming Chen, Yingqi Wei, Haiyang Chen, Lei He, Yi Zou, Xiaoxuan Long, Hongyu Zhan, Huaiming Wang, Hui Wang* and Yongjun Lu**

Supporting Information

Gut Microbiota-Derived 3-Hydroxybutyrate Blocks GPR43-Mediated IL6 Signaling to Ameliorate Radiation Proctopathy

Zhenhuang Ge, Chun Chen, Junyi Chen, Zhou Jiang, Lingming Chen, Yingqi Wei, Haiyang Chen, Lei He, Yi Zou, Xiaoxuan Long, Hongyu Zhan, Huaiming Wang*, Hui Wang* and Yongjun Lu*

Supplementary figures and legends

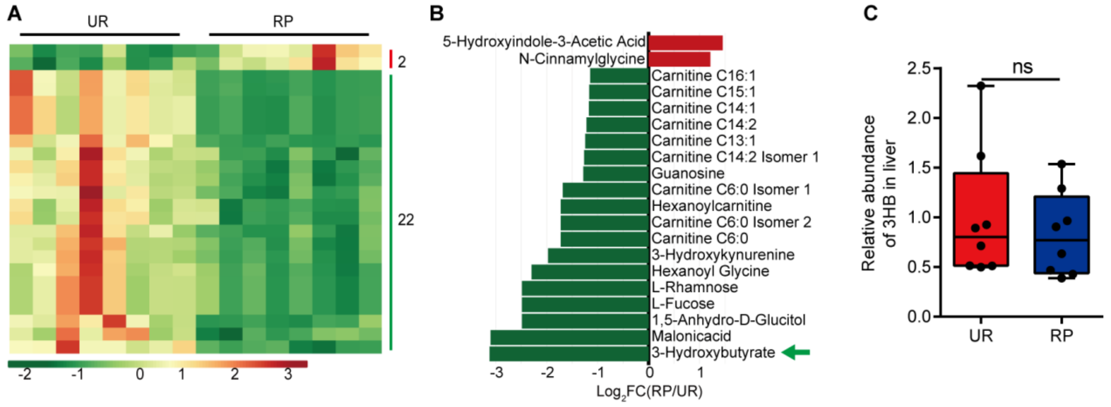

Figure S1. The concentrations of 3HB in serum that significantly decreased in RP mice are not related to liver.

(**A**) Heatmap of differentially enriched metabolites in serum samples between RP and UR mice.

(**B**) The top 20 significant differential metabolites in sera of RP and UR mice. Green arrowhead marks the 3HB.

(**C**) 3HB concentration shows no significant difference in liver after the treatment with radiation. ns, no significance, which is determined by the Student's *t*-test (C).

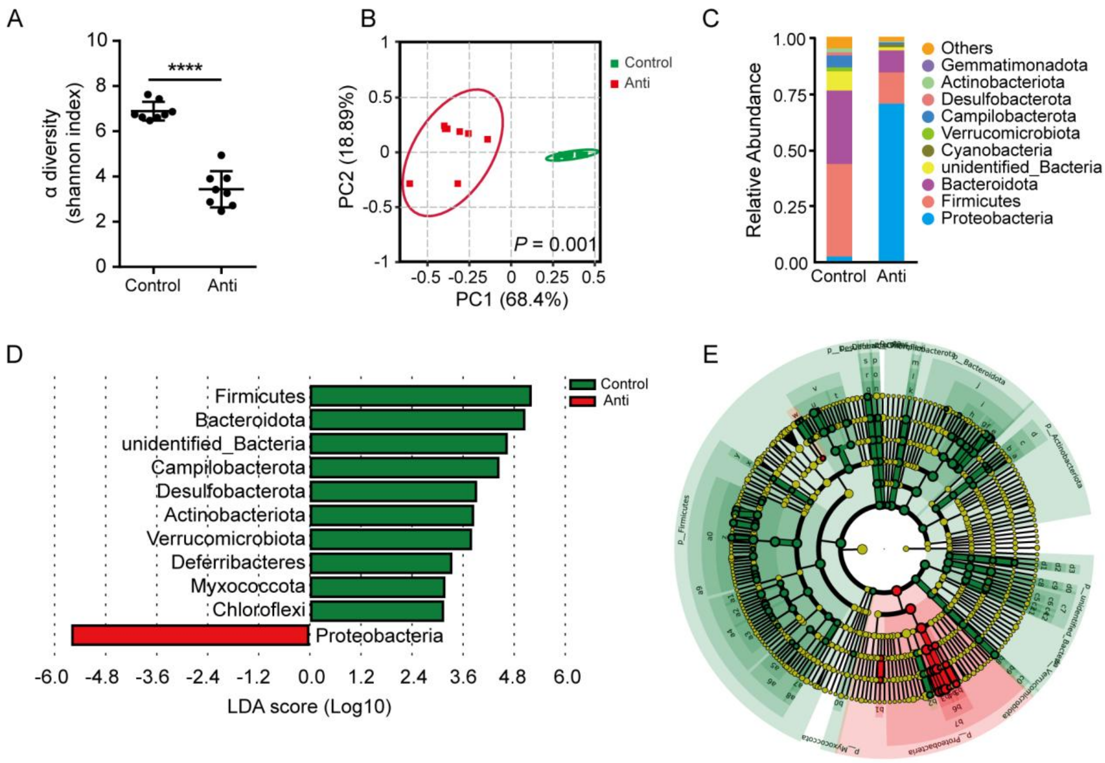

Figure S2. Antibiotic treatment removes most of the bacteria from the intestine.

(**A**) Alpha (α) diversity with Shannon index in mice with the treatment of antibiotics (Anti) compared to water (Control). ****P < 0.0001 determined by Student's t-test.

(**B**) PCoA plot (based on weighted UniFrac distances). P value is determined by the permutation multivariate analysis of variance (PERMANOVA) test.

(**C**) The relative abundance of gut bacteria at phylum level in the fecal samples.

(**D-E**) Histogram of the linear discriminant analysis (LDA) coupled with effect size measurements (LEfSe) [LDA significant threshold (log10) > ±3] (D) and cladogram tree (E) identified taxonomic biomarkers at phylum level between control and antibiotics treated mice. Higher abundant species in control mice (Control) and mice treated with antibiotics (Anti) are shaded in green and red, respectively.

Data are representative of at least two biological replicates. Data are presented as the mean ± SEM. Samples were collected after 6 weeks of antibiotic treatment. N = 8 per group.

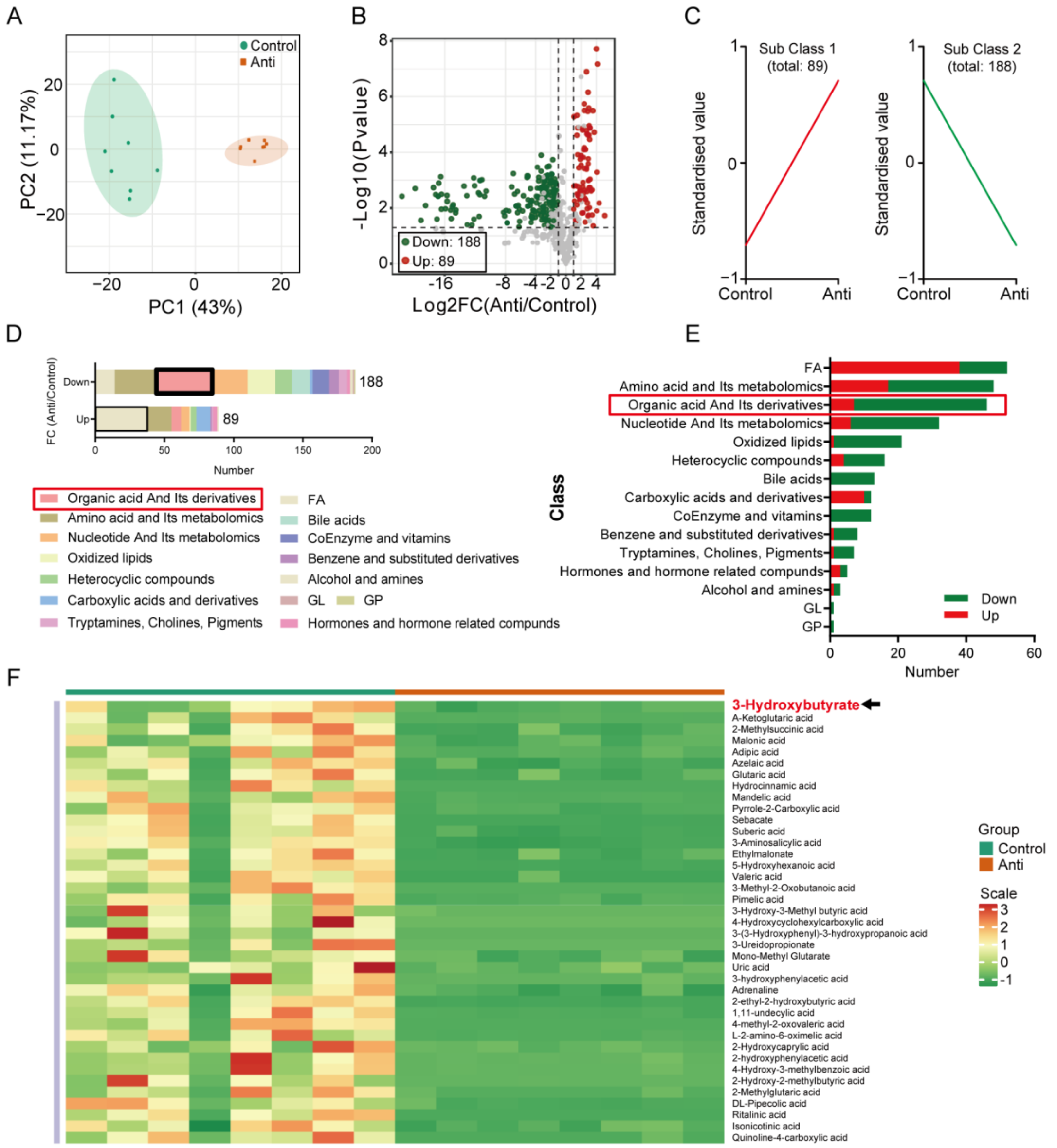

Figure S3. The concentration of many metabolites in feces is associated with antibiotic-removed gut bacteria, including organic acid and its derivatives with the most reduction in the absence of gut microbiota.

(**A**) PCA plot of feces metabolites from control mice (Control) and mice with antibiotics treatment (Anti).

(**B**) Volcano plot of all metabolites found in fecal samples. Red points indicate metabolites with a VIP (variable importance in projection) score > 1 and an adjusted $P < 0.05$ and log2(Anti/Control) > 1; green points indicate metabolites with a VIP score > 1 and an adjusted $P < 0.05$ and log2(Anti/Control) < -1.

(**C**) K-means analysis of the content change trend of metabolites in different samples.

(**D-E**) Cluster (D) and proportion (E) analysis of differentially enriched metabolites in feces between Control and Anti groups.

(**F**) Heatmap shows the significantly decreased metabolites in organic acids and their derivatives in fecal samples after antibiotic treatment.

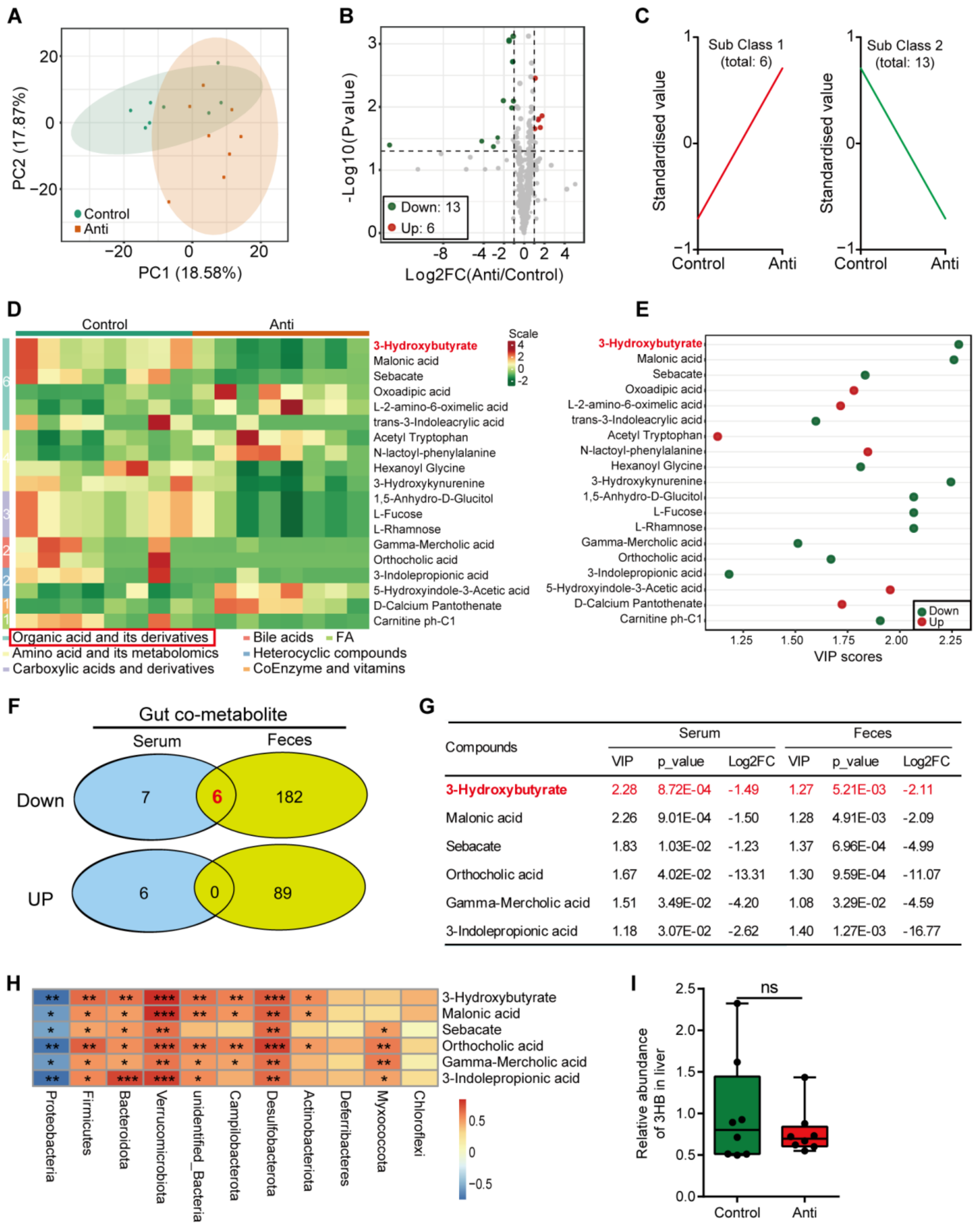

Compounds	Serum VIP	Serum p_value	Serum Log2FC	Feces VIP	Feces p_value	Feces Log2FC
**3-Hydroxybutyrate**	2.28	8.72E-04	-1.49	1.27	5.21E-03	-2.11
Malonic acid	2.26	9.01E-04	-1.50	1.28	4.91E-03	-2.09
Sebacate	1.83	1.03E-02	-1.23	1.37	6.96E-04	-4.99
Orthocholic acid	1.67	4.02E-02	-13.31	1.30	9.59E-04	-11.07
Gamma-Mercholic acid	1.51	3.49E-02	-4.20	1.08	3.29E-02	-4.59
3-Indolepropionic acid	1.18	3.07E-02	-2.62	1.40	1.27E-03	-16.77

Figure S4. The production of gut microbiota-derived 3HB in feces plays a dominant role in its concentration in serum.

(**A**) PCA plot of serum metabolites from control mice (Control) and mice with antibiotics treatment (Anti).

(**B**) Volcano plot of all metabolites found in serum samples. Red points indicate metabolites with a VIP (variable importance in projection) score > 1 and an adjusted

$P < 0.05$ and log2(Anti/Control) > 1; green points indicate metabolites with a VIP score > 1 and an adjusted $P < 0.05$ and log2(Anti/Control) < -1.

(**C**) K-means analysis of the content change trend of metabolites in different samples.

(**D**) Heatmap shows differentially enriched metabolites in sera between Control and Anti groups.

(**E**) VIP score of differential metabolites reveals their potential important role.

(**F-G**) Venn diagram (F) and list (G) display the six metabolites in sera that are directly associated with their feces concentration.

(**H**) Heatmap showing positive (red) and negative (blue) correlations between identified taxonomic biomarkers at phylum level (X axis) and fecal (Y axis) metabolites that are different in Control and Anti groups. *$P < 0.05$, **$P < 0.01$, and ***$P < 0.001$ determined by the Spearman correlation.

(**I**) The ability of the liver to produce 3HB is not different in UR mice pre-treated with or without antibiotics. ns, no significance, which is determined by the Student's *t*-test.

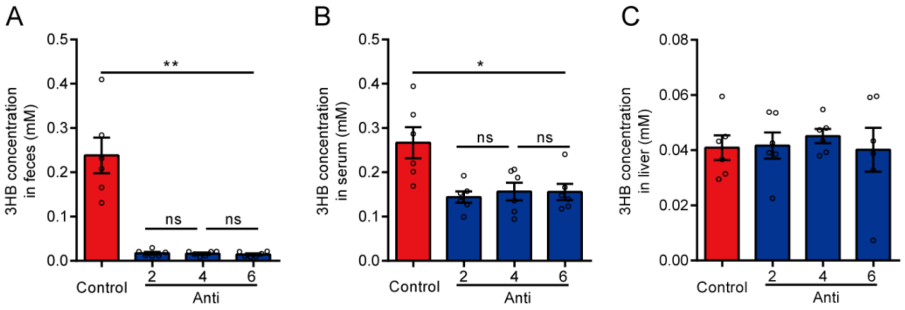

Figure S5. Longitudinal follow-up of 3HB concentrations after antibiotic treatment. (A) Fecal sample. (B) Serum sample. (C) Liver tissue.

Data are representative of at least two biological replicates. Data are presented as the mean ± SEM. Samples were collected after 2, 4, and 6 weeks of antibiotic treatment. N = 6 per group. *$P < 0.05$, and **$P < 0.01$ determined by the Student's *t*-test.

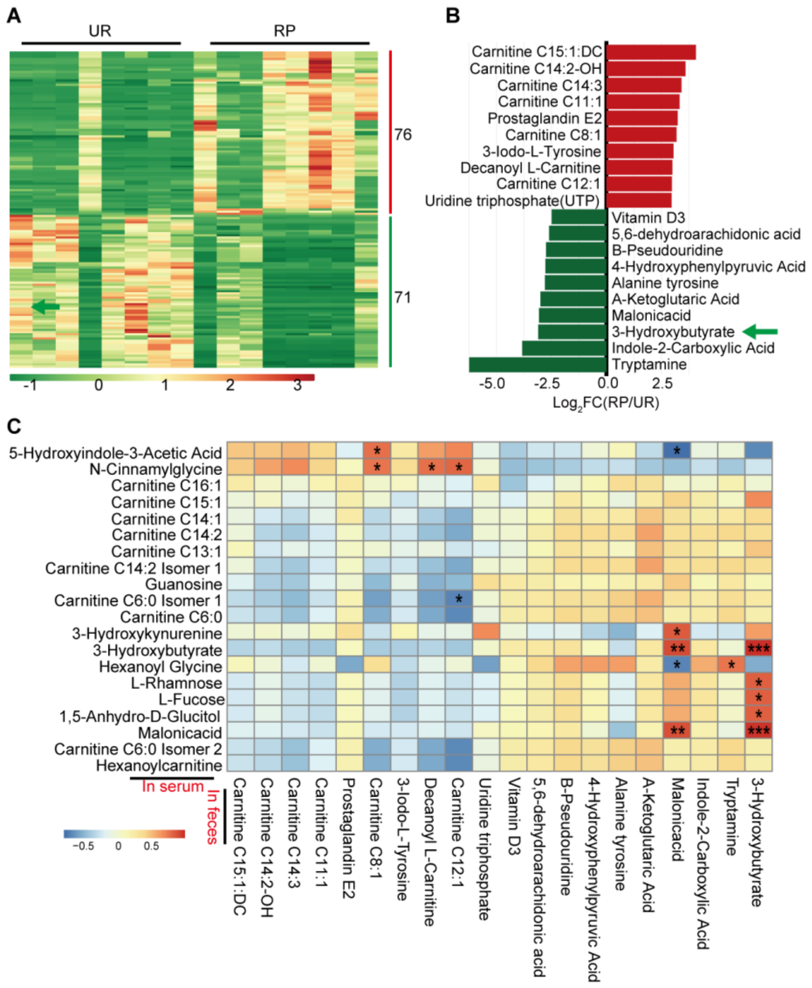

Figure S6. The concentrations of 3HB of stool and serum that significantly decreased in RP mice are positive correlation.

(**A**) Heatmap of enriched differential metabolites in fecal samples between RP and UR mice.

(**B**) The top 20 significant differential metabolites in feces of RP and UR mice. Green arrowhead marks the 3-hydroxybutyrate (3HB).

(**C**) Heatmap showing positive (red) and negative (blue) correlations between serum (X axis) and fecal (Y axis) metabolites measured in RP mice. **P* < 0.05, ***P* < 0.01, and ****P* < 0.001 determined by the Spearman correlation.

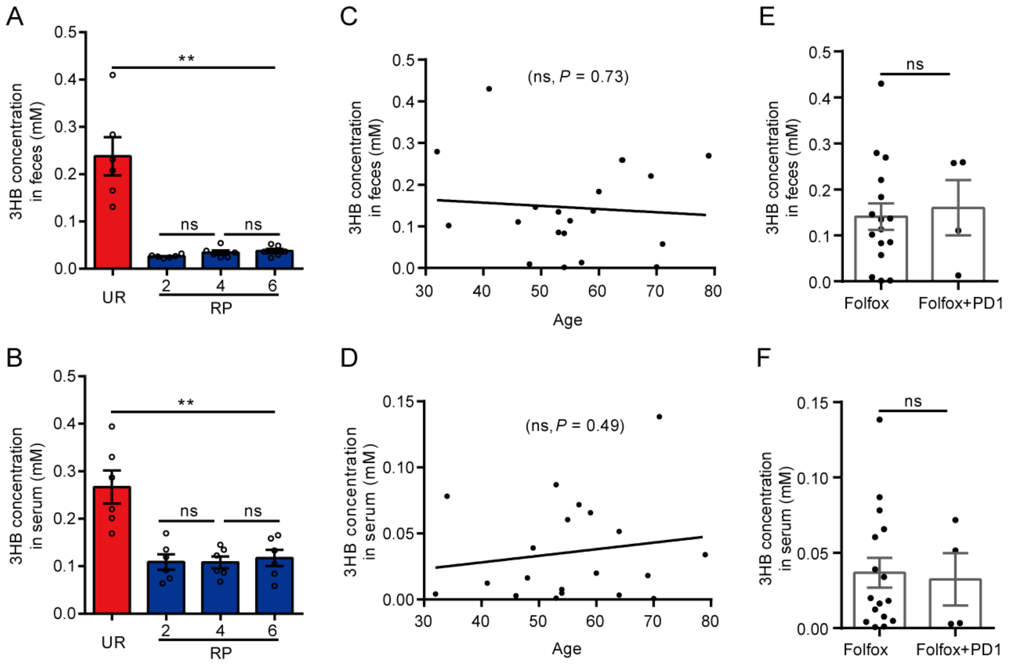

Figure S7. Longitudinal follow-up of 3HB and IL6 concentrations post-radiation.

(**A-B**) The concentration of 3HB in feces (A) and serum (B) sample derived from UR and RP mice.

(**C-D**) Correlation between age and 3HB concentration in feces (C) and serum (D) sample derived from oncology patients after radiotherapy treatment.

(**E-F**) The concentration of 3HB in feces (E) and serum (F) between oncology patients received with folfox and folfox/PD1 treatment.

Data are representative of at least two biological replicates. Data are presented as the mean ± SEM. Animal samples were collected at 2, 4, and 6 weeks respectively after radiation treatment, N = 6 per group. Patient samples were collected one day after the completion of radiotherapy (RT), N = 20 for oncology patients. $^{**}P < 0.01$ and no significance (ns) determined by the Student's *t*-test [(A), (B), (E), and (F)] and Spearman correlation [(C)and (D)].

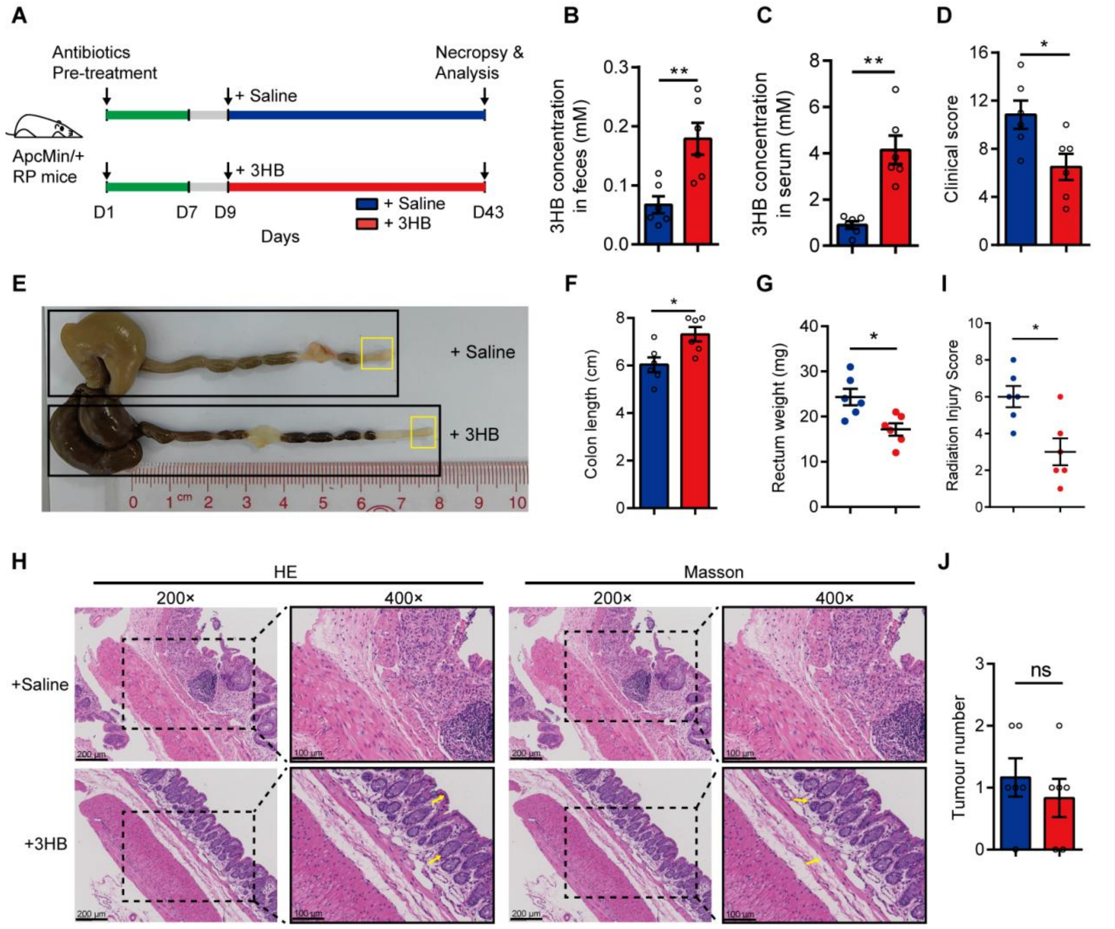

Figure S8. Oral administration of 3HB ameliorates radiation-induced damage but does not induce radioprotection of the tumor in colorectal cancer (CRC) mice post-radiation.

(**A**) Experimental diagram for determining the role of 3HB in CRC mice post-radiation. Construction of RP mouse model in APC$^{min/+}$ mice by radiation treatment. The models were then pre-treated with antibiotics for one week before orally administrated with 3HB (150 mg/kg body weight) or saline 3 times per week for 5 weeks.

(**B-C**) Concentrations of 3HB in fecal (B) and serum (C) samples.

(**D**) Clinical scores of the mice in each group.

(**E-G**) Representative images of the colorectal tissue (E) and colon length statistics (F). Boxed regions showed representative rectum images and rectum weight statistics (G).

(**H**) Representative images of H&E and Masson immunostaining of the distal rectum. Insets are showed at a higher magnification on the right.

(**I**) Histopathological changes evaluated by calculating RIS score.

(**J**) Tumor numbers in colorectal tissue of each group.

Data are representative of at least two biological replicates. Data are presented as the mean ± SEM. Samples were collected after 3HB treatment for 5 weeks. N = 6 per group. **P* < 0.05, and ***P* < 0.01 determined by the Student's *t*-test [(B), (C), (D), (F), (G), (I), and (J)].

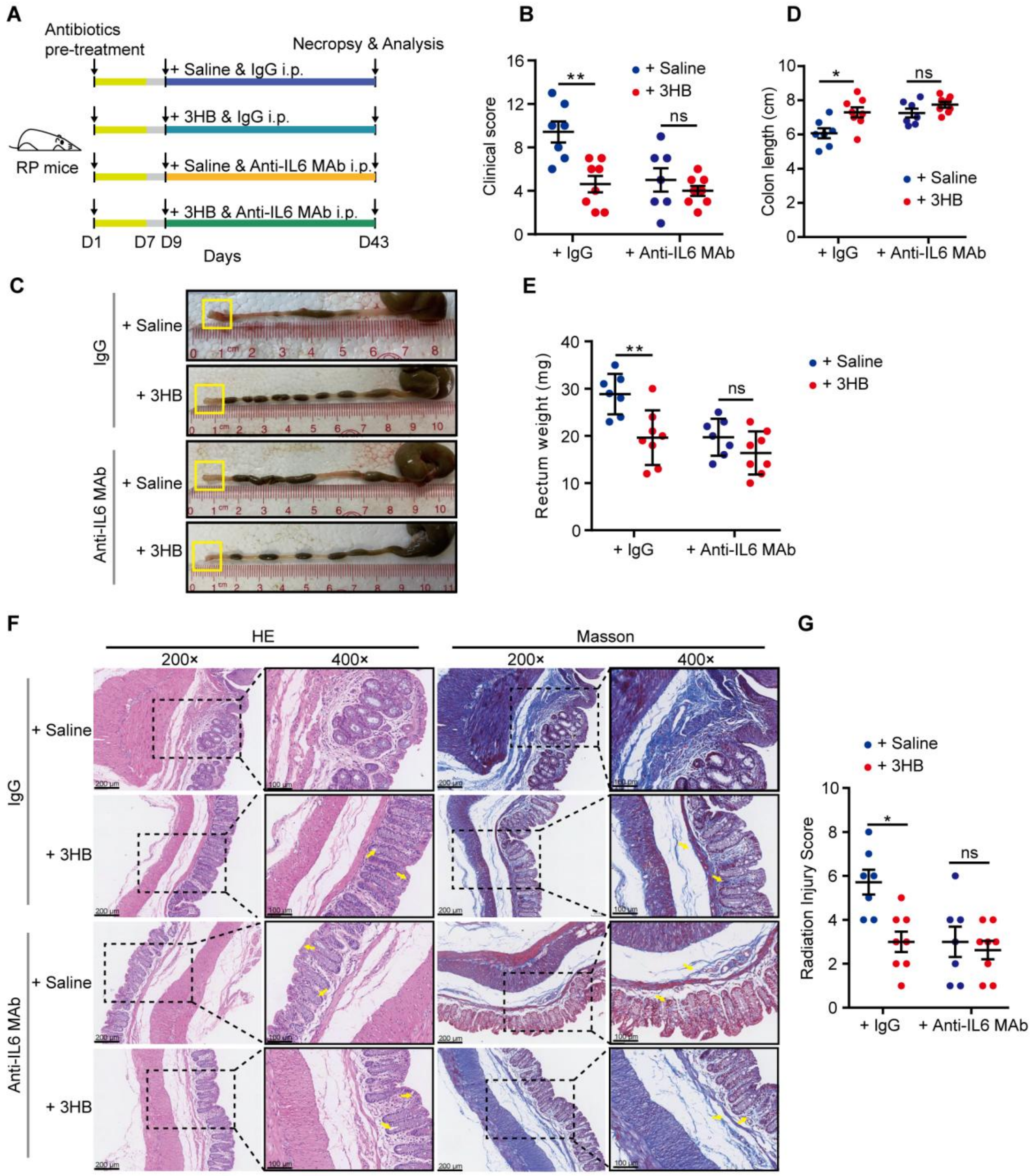

Figure S9. Reduced radiation-induced damage mediated by 3HB are impaired in the absence of IL6 signaling.

(**A**) Experimental diagram for determining whether the effect of 3HB depends on IL6 signaling in radiation-induced damage. Construction of RP mouse model by radiation treatment. RP mice were pre-treated with antibiotics for one week before injected with 5 mg/kg anti-IL6 MAb or IgG antibody control and orally administrated with 3HB (150 mg/kg body weight) or saline 3 times per week for 5 weeks.

(**B**) Clinical score for the mice in each group.

(**C-E**) Representative images of the colon (C) and colon length statistics (D). Boxed regions showed representative rectum images and rectum weight statistics (E).

(**F**) Representative images of H&E and Masson immunostaining of the distal rectums. Insets are demonstrated in higher magnification at right.

(**G**) Histopathological change evaluated by calculating RIS score.

Data are representative of at least two biological replicates. Data are presented as the mean ± SEM. Samples were collected after 3HB and anti-IL6 MAb treatment for 5 weeks, respectively. N = 7 for saline treated group; N = 8 for 3HB treated group. $^*P < 0.05$, and $^{**}P < 0.01$ determined by the Student's *t*-test [(B), (D), (E), and (G)].

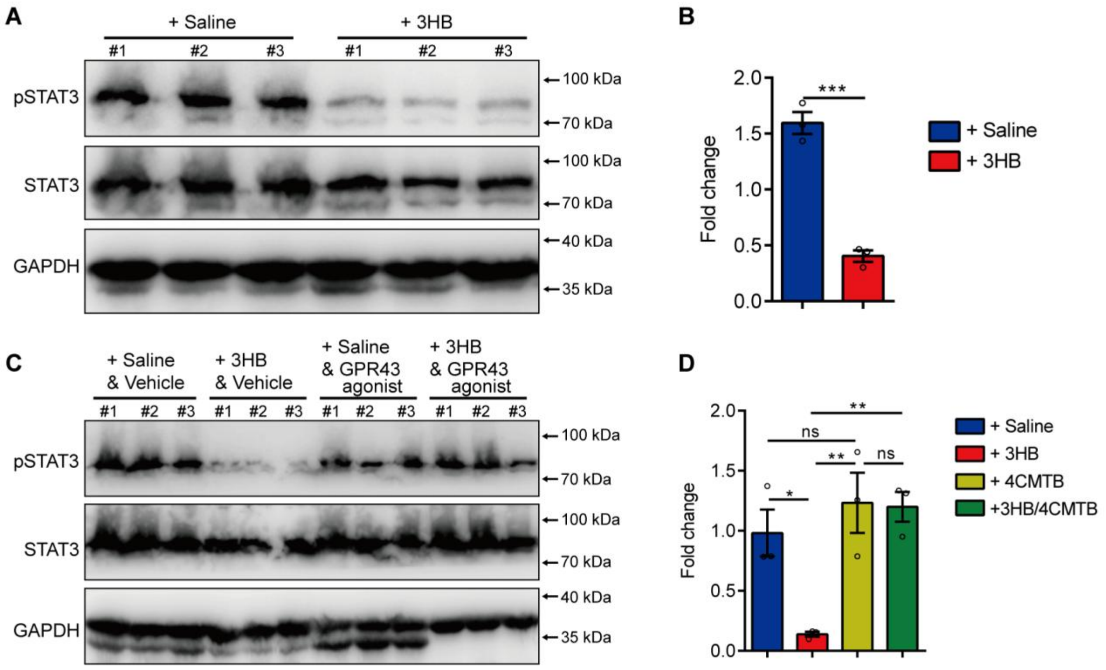

Figure S10. Western blot analysis of phosphorylated STAT3 in rectal tissue derived from RP mice with different treatments. Western blot for phosphorylated and total STAT3 in rectal tissue (left) and quantitative immunoblot analysis of pSTAT3 expression calculated by ImageJ (right). GAPDH served as a loading control. (**A-B**) RP mice pretreated with antibiotics for one week were orally administrated with 3HB (150 mg/kg body weight) or saline for 5 weeks (Figure 2).

(**C-D**) RP mice pre-treated with antibiotics were orally administrated with 3HB (150 mg/kg body weight) or saline for 5 weeks. Mice were injected with GPR43 agonist (4CMTB, 10 mg/kg body weight) or vehicle for 5 weeks at the same time (Figure 5).

N = 3 biologically independent samples. Data are presented as the mean ± SEM. *P < 0.05, **P < 0.01, and ***P < 0.001 determined by the Student's t-test (B) and one-way ANOVA with Tukey's multiple comparison test (D).

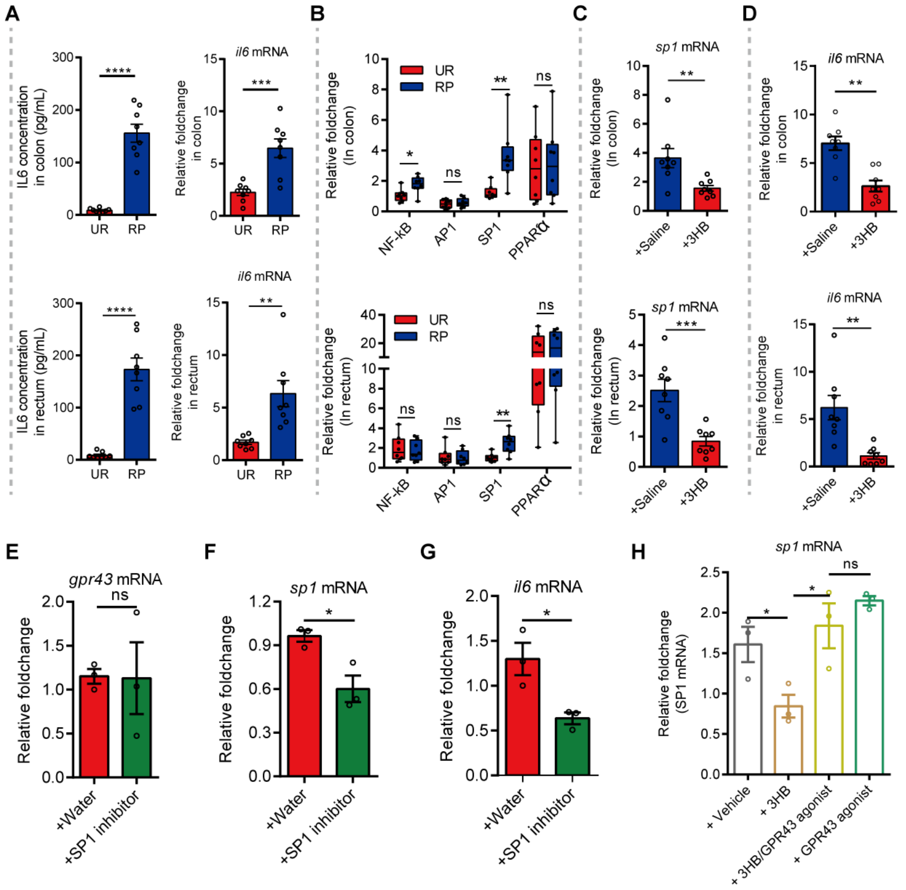

Figure S11. 3HB inhibits radiation-induced IL6 expression *via* transcriptional regulator SP1 mediated by GPR43.

(**A**) IL6 concentration and *il6* mRNA from colon and rectal tissue in UR and RP mice.

(**B**) The expression of known IL6 transcriptional regulators by quantitative PCR using mRNA extracted from the colon and rectal samples of UR and RP mice, respectively.

(**C-D**) The expression of *sp1* (C) and *il6* (D) from the colon and rectal samples in the RP mice treated with 3HB or saline.

(**E-G**) The expression of *gpr43* (E), *sp1* (F), and *il6* (G) in irradiated IECs treated with SP1 inhibitor (mithramycin, 25 nM).

(**H**) The expression of *sp1* in irradiated IECs treated with 3HB and GPR43 agonist.

Data are presented as the mean ± SEM. *$P < 0.05$, **$P < 0.01$, and ***$P < 0.001$ determined by the Student's *t*-test [(A), (B), (C), (D), (E), (F), and (G)] and one-way ANOVA with Tukey's multiple comparison test (H).

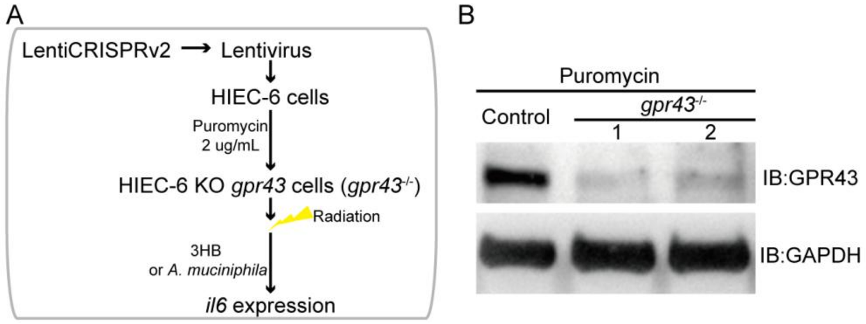

Figure S12. Knockout of *gpr43* in HIEC-6 intestinal epithelial cells.

(**A**) Experimental diagram for determining whether the regulation of 3HB and *A. muciniphila* is mediated by GPR43 in *gpr43*$^{-/-}$ cell line.

(**B**) The protein level of control and *gpr43*$^{-/-}$ cells confirmed the successful knockout of *gpr43* gene.

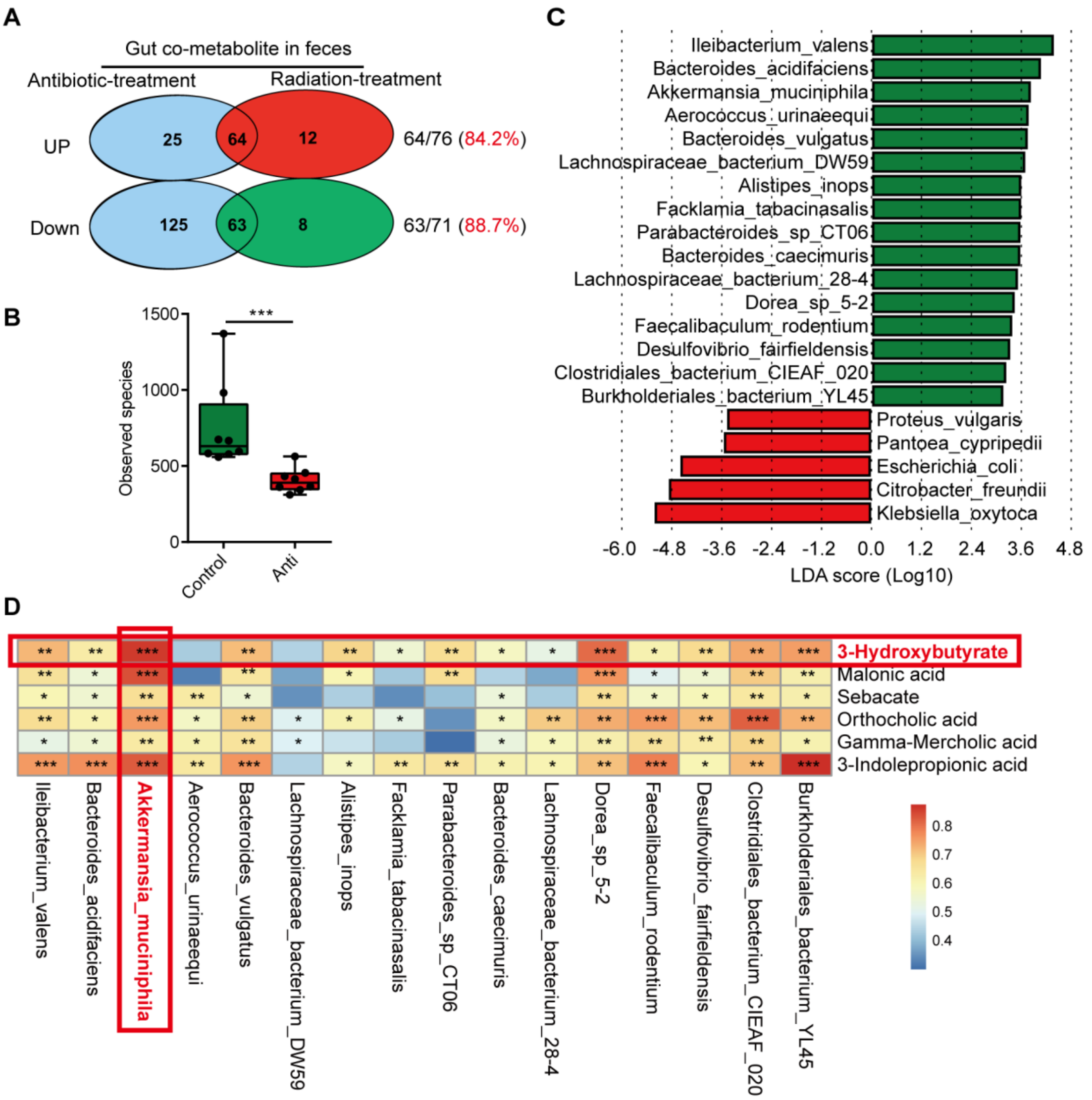

Figure S13. The concentration of 3HB in feces is associated with specific gut bacteria, in particularly with *A. muciniphila* abundance.

(**A**) Venn diagram displays that radiation-induced metabolite profile in feces is similar to that of antibiotic treatment, indicating the same alterations in gut microbiota.

(**B**) Observed species in control (Control) and antibiotic-treated (Anti) mice.

(**C**) Histogram of the linear discriminant analysis (LDA) coupled with effect size measurements (LEfSe) [LDA significant threshold (log10) > ±3] identified taxonomic biomarkers at species level between Control and Anti groups. Higher abundant species in Control and Anti are shaded in green and red, respectively.

(**D**) Heatmap showing positive and negative correlations between identified taxonomic biomarkers at species level (X axis) and six gut co-metabolites (Y axis) that are different in Control and Anti groups. The boxes in red frame indicate the significantly positive correlation between 3HB and *A. muciniphila*. $*P < 0.05$, $**P < 0.01$, and $***P < 0.001$ determined by Spearman correlation.

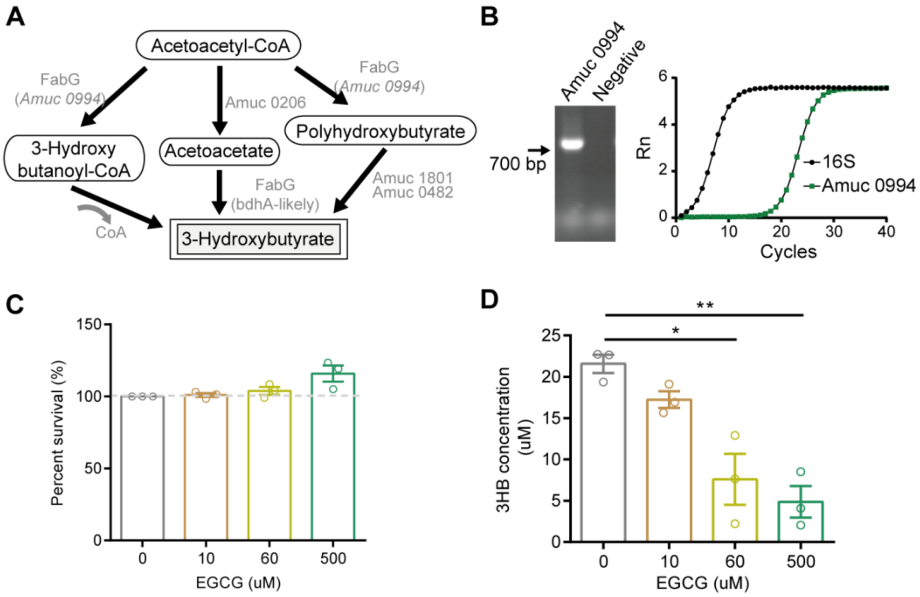

Figure S14. ***A. muciniphila*** **plays a contributing role in accumulating 3HB levels** ***via*** **FabG-mediated pathway.**

(**A**) Schematic diagram for 3HB biosynthesis pathway in bacteria according to Mierziak *et al*. FabG (Acetoacetyl-CoA reductase) can also function as PhaB.

(**B**) The existence and involvement of *fabG* gene (*Amuc 0994*) in 3HB biosynthesis pathway in *A. muciniphila* was confirmed through the bacterial genomic DNA and bacterial mRNA analysis.

(**C**) Survival of *A. muciniphila* bacteria in stationary phase treated with 0, 10, 60, and 500 μM EGCG for 24 hours.

(**D**) Measurement of 3HB concentration in the supernatant of *A. muciniphila* treated with 0, 10, 60, and 500 μM EGCG for 24 hours.

Data are representative of three independent biological replicates. Data are presented as the mean ± SEM. *$P < 0.05$ and **$P < 0.01$ determined by the Student's *t*-test (D).

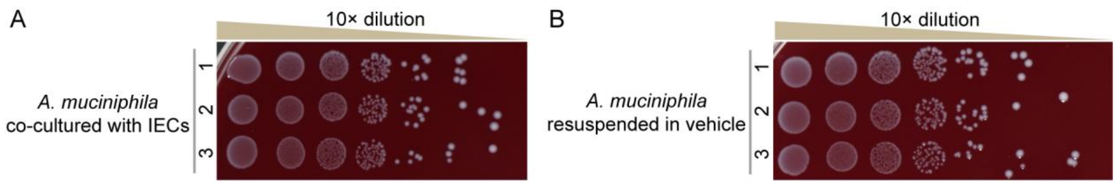

Figure S15. The viability of *A. muciniphila* in co-culture experiments.

(**A-B**) Viability of *A. muciniphila* resuspended in saline (A) or vehicle (B) after co-culturing with HIEC-6 cells for 24 hours. *A. muciniphila* can persists and does not proliferate during co-culture. The colonies were identified by PCR.

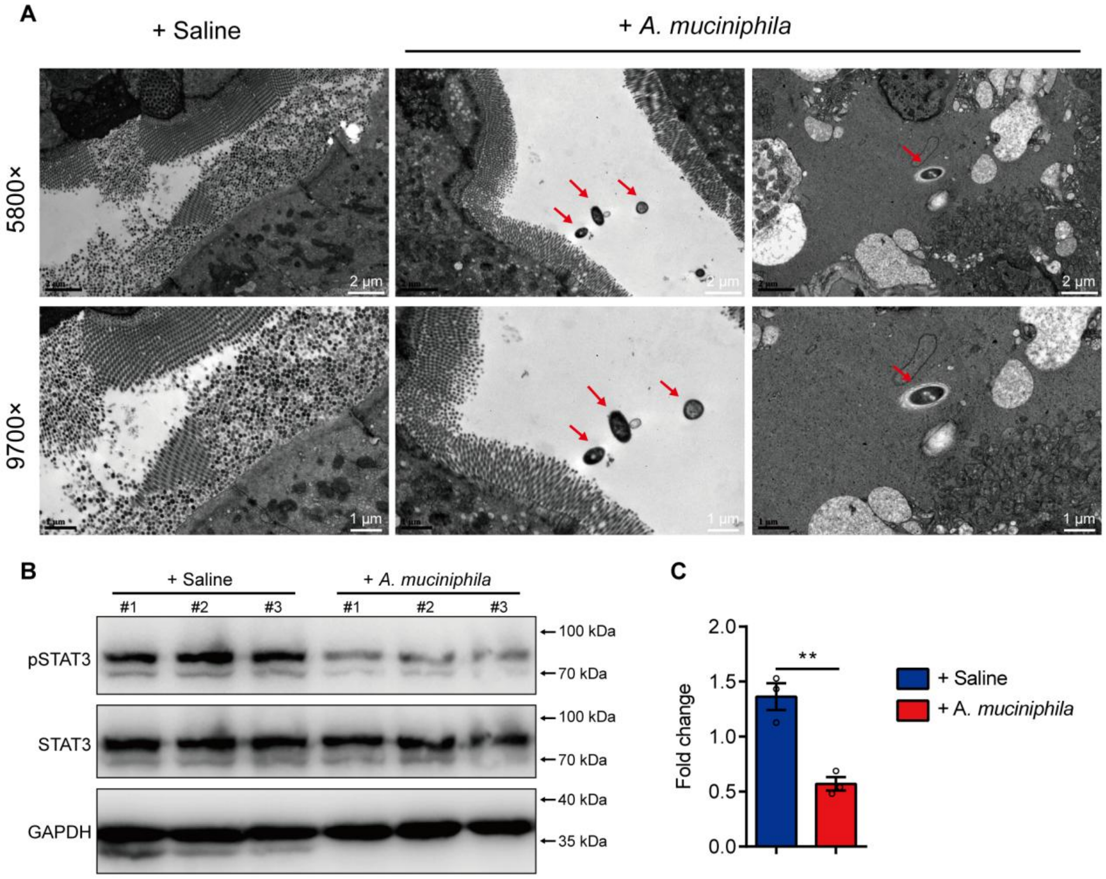

Figure S16. Oral administration of *A. muciniphila* can colonize the intestine of RP mice and downregulate the protein levels of pSTAT3 of rectal tissue.

(**A**) Representative transmission electron micrographs of intestinal samples from RP mice orally administrated with *A. muciniphila* or saline. Insets in higher magnification are in bottom row. *A. muciniphila* cells in the intestinal lumen and the intestines are observed at the left and the right panels, respectively, in mice orally administrated with *A. muciniphila*. Note that no or very few *A. muciniphila* is observed in mice treated with saline. Scale bars, 2 μm (Top panel) and 1 μm (Bottom panel). Red arrows indicate gut bacteria colonizing the colon tissues.

(**B-C**) RP mice pre-treated with antibiotics for one week were orally administrated with *A. muciniphila* (2×10^8) or saline for 5 weeks (Figure 7). N = 3 biologically independent samples. Data are presented as the mean ± SEM. $^{**}P < 0.01$ determined by the Student's *t*-test.

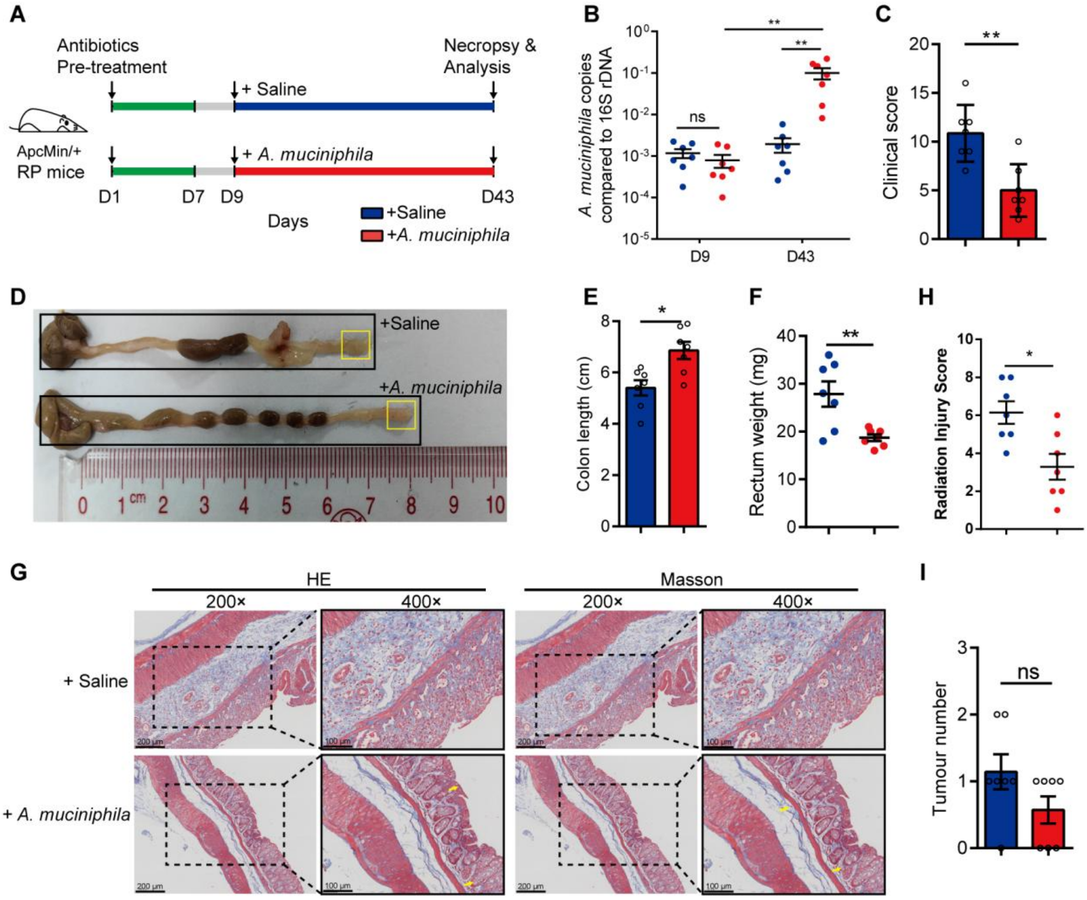

Figure S17. Gavage of *A. muciniphila* ameliorates radiation-induced damage but does not induce radioprotection of the tumor in CRC mice post-radiation.

(**A**) Experimental diagram for determining the role of *A. muciniphila* in CRC mice post-radiation. Construction of RP mouse model in $APC^{min/+}$ mice by radiation treatment. The models then were pre-treated with antibiotics for one week before orally administrated with *A. muciniphila* (2×10^8) or saline 3 times per week for 5 weeks.

(**B**) Analysis of *A. muciniphila* abundance in stool samples at indicated time points.

(**C**) Clinical scores of the mice in each group.

(**D-F**) Representative images of the colorectal tissue (E) and colon length statistics (F). Boxed regions showed representative rectum images and rectum weight statistics (G).

(**G**) Representative images of H&E and Masson immunostaining of the distal rectum. Insets are showed at a higher magnification on the right.

(**H**) Histopathological changes evaluated by calculating RIS score.

(**I**) Tumor numbers in colorectal tissue of each group.

Data are representative of at least two biological replicates. Data are presented as the mean ± SEM. Samples were collected after *A. muciniphila* treatment for 5 weeks. N = 7 per group. *$P < 0.05$, and **$P < 0.01$ determined by the Student's *t*-test [(B), (C), (E), (F), (H), and (I)].

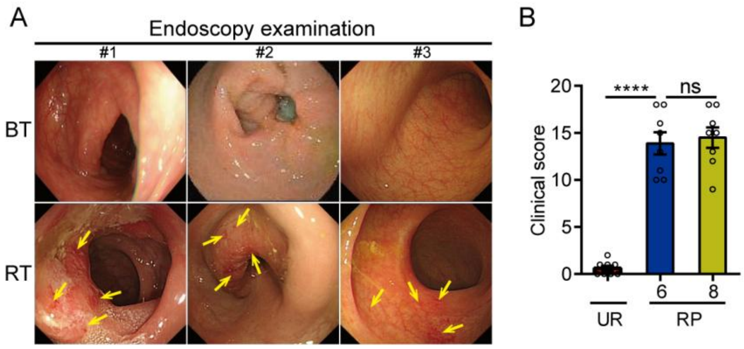

Figure S18. Examination of patients and mice after radiation therapy.

(**A**) Endoscopy examination confirms that patients had RP lesions after radiotherapy. Endoscopy examination showed subsequent changes in the rectal mucosa after a total irradiation dose of 50 Gy. The typical case revealed telangiectasis and active bleeding sites in the intestinal mucosa within the RP lesions (yellow arrow).

(**B**) Clinical score of mice before radiation and post-radiation at 6 and 8 weeks.

Supplementary Tables

Table S1. Radiation Injury Score (RIS) Parameters for radiation proctopathy.

Assess the following parameters:	
A. Mucosal ulceration	
	0 = NO ulcerations in mucosa
	1 = Small superficial ulcerations
	2 = Ulcerations involving submucosa
B. Inflammatory cell infiltrate	
	0 = Normal
	1 = Increased density of inflammation cells with focal aggregation in mucosa or submucosa
	2 = Multi-focal aggregation of inflammatory cells in mucosa or submucosa
C. Edema	
	0 = NO edema
	1 = Edema
D. Vascular stenosis	
	0 = Normal
	1 = 25% - 49% stenosis
	2 = 50% - 75% stenosis
	3 = More than 75% stenosis
E. Submucosa fibrosis	
	0 = Normal
	1 = Mild increase in collagen fibers
	2 = Dense fibers were significantly increased, and the vessel wall was hyaline degeneration

Table S2. The significant expressed metabolites in serum between RP and UR (RP to UR)

Formula	Compounds	VIP	P value	Fold Change	Log2FC (RP/UR)	Type
C6H12O5	1,5-Anhydro-D-Glucitol	2.38	0.00007	0.18	-2.48	down
C6H12O5	L-Fucose	2.38	0.00007	0.18	-2.48	down
C6H12O5	L-Rhamnose	2.38	0.00007	0.18	-2.48	down
C4H8O3	3-Hydroxybutyrate	2.40	0.00016	0.12	-3.12	down
C3H4O4	Malonicacid	2.38	0.00017	0.12	-3.10	down
C4H7NO3	N-Acetylglycine	2.03	0.00114	0.49	-1.02	down
C11H11NO3	N-Cinnamylglycine	2.05	0.00148	2.31	1.21	up
C8H15NO3	Hexanoyl Glycine	2.24	0.00042	0.20	-2.30	down
C10H13N5O5	Guanosine	2.34	0.00005	0.41	-1.28	down
C10H9NO3	5-Hydroxyindole-3-Acetic Acid	1.99	0.00202	2.73	1.45	up
C10H12N2O4	3-Hydroxykynurenine	2.45	0.00008	0.25	-1.97	down
C13H25NO4	Hexanoylcarnitine	2.13	0.00200	0.30	-1.72	down
C23H43NO4	Carnitine C16:1	2.02	0.00178	0.45	-1.14	down
C23H41NO4	Carnitine C16:2	2.21	0.00034	0.47	-1.09	down
C22H41NO4	Carnitine C15:1	2.17	0.00114	0.45	-1.16	down
C21H39NO4	Carnitine C14:1	1.99	0.00177	0.44	-1.17	down
C21H37NO4	Carnitine C14:2	2.12	0.00148	0.43	-1.22	down
C20H37NO4	Carnitine C13:1	2.17	0.00093	0.42	-1.24	down
C13H25NO4	Carnitine C6:0 Isomer 2	2.13	0.00200	0.30	-1.72	down
C13H25NO4	Carnitine C6:0 Isomer 1	2.13	0.00126	0.31	-1.68	down
C13H25NO4	Carnitine C6:0	2.13	0.00200	0.30	-1.72	down
C21H37NO4	Carnitine C14:2 Isomer 1	2.15	0.00175	0.42	-1.27	down
C23H41NO4	Carnitine C16:2 Isomer1	2.24	0.00032	0.46	-1.13	down
C21H35NO6	Carnitine C14:2:DC	2.03	0.00124	0.46	-1.13	down

Table S3. The significant expressed metabolites in feces between RP and UR (RP to UR)

Formula	Compounds	VIP	P value	Fold Change	Log2FC (RP/UR)	Type
C6H13N3O3	L-Citrulline	1.44	0.00620	0.256	-1.97	down
C9H8O4	4-Hydroxyphenylpyruvic Acid	1.11	0.03252	0.146	-2.77	down
C11H20N2O6	L-Saccharopine	1.39	0.00730	4.752	2.25	up
C13H16N2O4	Phenylacetyl-L-Glutamine	1.34	0.01802	2.505	1.32	up
C3H7NO5S2	S-Sulfo-L-Cysteine	1.19	0.04188	5.786	2.53	up
C6H5NO2	2-Picolinic Acid	1.45	0.02000	0.470	-1.09	down
C5H4N4O2	Xanthine	1.20	0.02965	0.457	-1.13	down
C9H12N2O5	2'-Deoxyuridine	1.29	0.01142	0.274	-1.87	down
C10H13N5O6	8-Hydroxyguanosine	1.09	0.04069	3.231	1.69	up
C9H14N3O8P	Cytidine-5-Monophosphate	1.33	0.00484	0.418	-1.26	down
C27H33N9O15P2	Flavin Adenine Dinucleotide	1.25	0.02747	0.299	-1.74	down
C5H4N4O	Hypoxanthine	1.51	0.01386	0.355	-1.49	down
C10H14N2O5	Thymidine	1.18	0.03644	0.365	-1.45	down
C15H12I3NO4	3,3',5-Triiodo-L-Thyronine	1.43	0.00270	0.367	-1.45	down
C5H6O5	A-Ketoglutaric Acid	1.58	0.00908	0.125	-3.00	down
C6H12O6	D-Glucose	1.05	0.02573	2.279	1.19	up
C18H32O16	D-Melezitose	1.18	0.01569	2.752	1.46	up
C18H32O16	Maltotriose	1.18	0.01569	2.752	1.46	up
C27H44O	Vitamin D3	1.47	0.00230	0.178	-2.49	down
C9H17NO5	Pantothenate	1.25	0.03200	0.409	-1.29	down
C9H7NO2	Indole-2-Carboxylic Acid	1.11	0.01664	0.070	-3.83	down
C5H8O4	2-Methylsuccinic Acid	1.31	0.01042	0.281	-1.83	down
C4H8O3	3-Hydroxybutyrate	1.69	0.00249	0.116	-3.10	down
C9H16O4	Azelaic Acid	1.49	0.00361	0.399	-1.33	down
C12H22O4	Dodecanedioic Aicd	1.44	0.00899	0.190	-2.40	down
C5H8O4	Glutaric Acid	1.31	0.01042	0.281	-1.83	down
C3H9N3O3S	Guanidinoethyl Sulfonate	1.23	0.01161	3.896	1.96	up
C9H10O2	Hydrocinnamic Acid	1.28	0.01665	0.226	-2.14	down
C4H6O6	L-Tartaric Acid	1.35	0.04232	4.781	2.26	up
C3H4O4	Malonicacid	1.68	0.00231	0.119	-3.07	down
C10H18O4	Sebacate	1.33	0.00944	0.294	-1.77	down
C8H14O4	Subericacid	1.24	0.02053	0.442	-1.18	down
C20H32O5	LipoxinA4 [5S,6R,15S-trihydroxy-7E,9E,11Z,13E-eicosatetraenoic acid]	1.22	0.01443	0.371	-1.43	down
C20H34O2	Cis-11,14,17-Eicosatrienoic Acid(C20:3)	1.27	0.04072	0.434	-1.20	down
C20H40O2	Arachidic Acid(C20:0)	1.31	0.01330	0.324	-1.63	down
C5H8O4	Ethylmalonate	1.31	0.01042	0.281	-1.83	down
C12H17N5O5	2-(Dimethylamino)Guanosine	1.42	0.01703	4.838	2.27	up
C6H12O3	5-Hydroxyhexanoic Acid	1.24	0.02507	0.362	-1.47	down
C10H13N5O5	8-Hydroxy-2-Deoxyguanosine	1.37	0.02962	3.411	1.77	up

C9H12N2O6	B-Pseudouridine	1.61	0.02294	0.149	-2.75	down
C6H8O5	Oxoadipic Acid	1.24	0.01557	0.284	-1.82	down
C5H10O2	Valeric Acid	1.44	0.01103	0.261	-1.94	down
C5H8O3	3-Methyl-2-Oxobutanoic Acid	1.27	0.01594	0.190	-2.40	down
C11H11NO3	Indole-3-lactic acid	1.79	0.00351	3.352	1.75	up
C6H10O8	D-Glucarate	1.16	0.03119	4.009	2.00	up
C10H12N4O6	Xanthosine	1.30	0.03727	0.282	-1.82	down
C20H32O6	6-Ketoprostaglandin E1	1.16	0.04539	5.393	2.43	up
C5H9NO3	N-Acetyl-L-alanine	1.15	0.02409	2.206	1.14	up
C9H15N2O15P3	Uridine triphosphate(UTP)	1.17	0.03781	7.773	2.96	up
C18H34O3	Ricinoleic acid	1.03	0.04533	0.441	-1.18	down
C20H34O4	5,6-DiHETrE [(±)5,6-dihydroxy-8Z,11Z,14Z-eicosatrienoic acid]	1.08	0.04002	0.469	-1.09	down
C20H34O5	5-iPF2α-VI [(8β)-5,9α,11α-trihydroxy-prosta-6E,14Z-dien-1-oic acid]	1.13	0.01101	0.340	-1.56	down
C20H32O5	Prostaglandin E2	1.17	0.04554	9.426	3.24	up
C5H9NO3	N-acetyl-beta-alanine	1.18	0.02353	2.221	1.15	up
C15H12I3NO4	3,3',5'-Triiodothyronine	1.43	0.00270	0.367	-1.45	down
C26H43NO5	Glycine deoxycholic acid	1.22	0.04484	0.274	-1.87	down
C18H32O4	13-HpODE	1.31	0.01450	0.331	-1.60	down
C6H12O3	2-ethyl-2-hydroxybutyric acid	1.25	0.02073	0.352	-1.51	down
C24H38O4	7-ketolithocholic acid	1.20	0.00585	0.253	-1.98	down
C13H24O4	1,11-undecylic acid	1.32	0.02050	0.381	-1.39	down
C16H32O3	16-Hydroxyhexadecanoic acid	1.36	0.00677	0.338	-1.56	down
C24H38O4	12-ketolithocholic acid	1.20	0.00585	0.253	-1.98	down
C19H40O3	Heparin	1.53	0.00140	0.208	-2.26	down
C24H38O4	Orthocholic acid	1.20	0.00430	0.233	-2.10	down
C9H7NO2	Indole-3-carboxylic acid	1.39	0.04221	0.235	-2.09	down
C6H10O3	4-methyl-2-oxovaleric acid	1.27	0.01559	0.239	-2.07	down
C10H13NO6S	L-tyrosine methyl ester 4-sulfate	1.20	0.02523	4.851	2.28	up
C5H9NO3	2-amino-4-oxovaleric acid	1.18	0.02353	2.221	1.15	up
C10H11NO4	P-hydroxyphenylacetylglycine	1.09	0.02861	2.984	1.58	up
C7H11NO5	L-2-amino-6-oximelic acid	1.44	0.01650	0.291	-1.78	down
C6H10O8	Mucic Acid	1.15	0.03004	4.478	2.16	up
C3H7NO2	L-Alanine	1.78	0.00068	0.357	-1.49	down
C9H10INO3	3-Iodo-L-Tyrosine	1.17	0.02436	8.275	3.05	up
C11H12N2O3	5-Hydroxy-L-Tryptophan	1.03	0.02035	5.944	2.57	up
C5H7NO3	5-Oxoproline	1.29	0.01309	0.398	-1.33	down
C6H9NO5	N-Acetylaspartate	1.65	0.00523	4.311	2.11	up
C7H11NO5	N-Acetyl-L-Glutamic Acid	1.24	0.00672	0.208	-2.26	down
C8H15NO6	N-Acetylmannosamine	1.21	0.01537	0.379	-1.40	down
C5H14NO+	Choline	1.39	0.04739	2.800	1.49	up
C5H6N2O2	Thymine	1.50	0.01806	0.236	-2.08	down

C15H11I4NO4	L-Thyroxine	1.25	0.02398	0.260	-1.94	down
C10H12N2	Tryptamine	1.37	0.01332	0.013	-6.24	down
C6H5NO2	Nicotinic Acid	1.50	0.00395	0.361	-1.47	down
C17H20N4O6	Riboflavin	1.25	0.01547	0.482	-1.05	down
C4H7N3O	Creatinine	1.53	0.02293	6.422	2.68	up
C13H16N2O4	N-γ-Acetyl-N-2-Formyl-5-Methoxykynurenamine	1.85	0.00032	3.570	1.84	up
C9H11N5O3	Biopterin	1.47	0.02550	3.458	1.79	up
C8H12NO6P	Pyridoxine 5'-Phosphate	1.54	0.01343	0.219	-2.19	down
C6H11NO2	DL-Pipecolic Acid	1.35	0.00825	0.385	-1.38	down
C10H7NO4	Xanthurenic Acid	1.57	0.00349	0.203	-2.30	down
C6H11NO4	2-Aminoadipic Acid	1.41	0.00999	0.489	-1.03	down
C20H23N7O7	10-Formyl-Thf	1.65	0.00502	7.241	2.86	up
C6H13NO2	L-Norleucine	1.79	0.00430	2.666	1.41	up
C10H10N2O	Indole-3-acetamide	1.42	0.01916	5.036	2.33	up
C18H28O2	Stearidonic Acid	1.26	0.03314	0.415	-1.27	down
C12H23NO4	2-Methylbutyroylcarnitine	1.51	0.01147	4.287	2.10	up
C21H42NO7P	LysoPE(16:1(9Z)/0:0)	1.56	0.03205	2.265	1.18	up
C6H10N2O4	N-Alpha-Acetyl-L-Asparagine	1.41	0.01755	3.602	1.85	up
C3H7NO2	β-Alanine	1.78	0.00068	0.357	-1.49	down
C33H34N4O6	Biliverdin	1.47	0.00799	3.259	1.70	up
C13H15N3O3	Glycyl-tryptophan	1.38	0.03965	3.002	1.59	up
C5H4N4O2	Oxypurinol	1.47	0.00125	0.270	-1.89	down
C19H37NO4	Dodecylcarnitine	1.62	0.01302	5.845	2.55	up
C17H33NO4	Decanoyl L-Carnitine	1.49	0.04753	7.966	2.99	up
C24H40O3	Lithocholic acid	1.20	0.01503	0.213	-2.23	down
C15H12O5	(-)-Norepinephrine	1.72	0.00200	3.994	2.00	up
C20H30O2	5,6-dehydroarachidonic acid	1.23	0.04213	0.164	-2.61	down
C21H41NO4	(±) -Myristylcarnitine	1.44	0.02032	3.745	1.90	up
C20H23N7O7	Folic acid	1.65	0.00502	7.241	2.86	up
C33H42N4O6	Urobilin	1.21	0.02975	0.353	-1.50	down
C13H25NO4	Hexanoylcarnitine	1.40	0.04836	4.791	2.26	up
C12H16N2O4	Alanine tyrosine	1.47	0.00136	0.144	-2.80	down
C25H45NO5	Carnitine C18:2-OH	1.72	0.00651	3.373	1.75	up
C25H45NO4	Carnitine C18:2	1.34	0.03225	2.072	1.05	up
C25H43NO4	Carnitine C18:3	1.36	0.02650	2.889	1.53	up
C25H41NO4	Carnitine C18:4	1.67	0.01768	6.272	2.65	up
C22H39NO6	Carnitine C15:1:DC	1.62	0.00440	16.743	4.07	up
C23H43NO4	Carnitine C16:1	1.50	0.01568	2.896	1.53	up
C23H41NO4	Carnitine C16:2	1.51	0.01866	7.723	2.95	up
C23H39NO4	Carnitine C16:3	1.53	0.01561	5.221	2.38	up
C21H41NO5	Carnitine C14-OH	1.75	0.00514	3.433	1.78	up
C21H39NO5	Carnitine C14:1-OH	1.34	0.00328	6.599	2.72	up
C22H41NO4	Carnitine C15:1	1.20	0.01203	4.757	2.25	up
C21H37NO5	Carnitine C14:2-OH	1.59	0.01481	12.059	3.59	up

C21H41NO4	Carnitine C14:0	1.42	0.01798	3.991	2.00	up
C21H39NO4	Carnitine C14:1	1.61	0.00867	6.792	2.76	up
C21H37NO4	Carnitine C14:2	1.31	0.02474	7.756	2.96	up
C21H35NO4	Carnitine C14:3	1.40	0.04707	10.653	3.41	up
C19H37NO5	Carnitine C12-OH	1.49	0.00576	5.981	2.58	up
C18H33NO6	Carnitine C11:DC	1.49	0.00609	5.844	2.55	up
C20H39NO4	Carnitine C13:0	1.80	0.00478	5.668	2.50	up
C20H37NO4	Carnitine C13:1	1.72	0.00338	2.987	1.58	up
C19H35NO4	Carnitine C12:1	1.65	0.01253	7.877	2.98	up
C18H35NO4	Carnitine C11:0	1.24	0.00926	6.785	2.76	up
C18H33NO4	Carnitine C11:1	1.74	0.02537	10.002	3.32	up
C17H33NO4	Carnitine C10:0	1.53	0.04268	7.760	2.96	up
C15H29NO5	Carnitine C8-OH	1.41	0.02806	4.641	2.21	up
C16H31NO4	Carnitine C9:0	1.90	0.01021	6.693	2.74	up
C15H27NO4	Carnitine C8:1	1.51	0.02298	9.103	3.19	up
C13H25NO4	Carnitine C6:0	1.40	0.04836	4.791	2.26	up
C12H23NO4	Carnitine C5:0	1.50	0.00995	4.235	2.08	up
C8H15NO6	N-Acetyl-D-Galactosamine	1.15	0.01998	0.451	-1.15	down
C4H4N6O	8-Azaguanine	1.47	0.00355	0.271	-1.88	down
C19H31NO4	Carnitine C12:3	1.42	0.01414	6.831	2.77	up
C21H35NO6	Carnitine C14:2:DC	1.48	0.01660	2.838	1.50	up
C23H41NO5	Carnitine C16:2-OH	1.54	0.00357	7.769	2.96	up
C23H41NO5	Carnitine C16:2-OH Isomer 1	1.67	0.00243	7.700	2.94	up

Table S4. The details of correlation between serum and fecal metabolites measured in the RP mice.

Spearman correlation between serum and fecal metabolites measured in the RP mice.

	Carnitine C15:1:DC	Carnitine C14:2-OH	Carnitine C14:3	Carnitine C11:1	Prostaglandin E2	Carnitine C8:1	3-Iodo-L-Tyrosine	Decanoyl L-Carnitine	Carnitine C12:1	Uridine triphosphate
5-Hydroxyindole-3-Acetic Acid	R = 0.45 *P* = 0.26	R = 0.45 *P* = 0.26	R = 0.50 *P* = 0.20	R = 0.38 *P* = 0.35	R = -0.21 *P* = 0.61	R = 0.76 *P* = 0.02	R = 0.24 *P* = 0.56	R = 0.59 *P* = 0.11	R = 0.69 *P* = 0.06	R = -0.14 *P* = 0.72
N-Cinnamylglycine	R = 0.42 *P* = 0.28	R = 0.57 *P* = 0.13	R = 0.65 *P* = 0.07	R = 0.40 *P* = 0.31	R = 0.07 *P* = 0.86	R = 0.73 *P* = 0.03	R = 0.34 *P* = 0.40	R = 0.76 *P* = 0.02	R = 0.78 *P* = 0.02	R = -0.04 *P* = 0.90
Carnitine C16:1	R = 0.21 *P* = 0.61	R = 0.02 *P* = 0.95	R = -0.04 *P* = 0.91	R = 0.14 *P* = 0.73	R = 0.26 *P* = 0.53	R = 0 *P* = 1	R = 0.04 *P* = 0.90	R = -0.11 *P* = 0.77	R = -0.21 *P* = 0.61	R = 0.24 *P* = 0.56
Carnitine C15:1	R = -0.07 *P* = 0.86	R = 0.02 *P* = 0.95	R = -0.17 *P* = 0.67	R = -0.11 *P* = 0.77	R = 0.19 *P* = 0.65	R = -0.02 *P* = 0.95	R = -0.26 *P* = 0.52	R = -0.11 *P* = 0.77	R = -0.21 *P* = 0.61	R = -0.07 *P* = 0.86
Carnitine C14:1	R = -0.09 *P* = 0.82	R = -0.23 *P* = 0.57	R = -0.32 *P* = 0.43	R = -0.14 *P* = 0.73	R = 0.21 *P* = 0.61	R = -0.33 *P* = 0.41	R = -0.17 *P* = 0.68	R = -0.38 *P* = 0.35	R = -0.52 *P* = 0.18	R = -0.02 *P* = 0.95
Carnitine C14:2	R = -0.07 *P* = 0.86	R = -0.35 *P* = 0.38	R = -0.41 *P* =0.30	R = -0.23 *P* = 0.57	R = 0.04 *P* = 0.91	R = -0.30 *P* = 0.45	R = -0.26 *P* = 0.52	R = -0.45 *P* = 0.26	R = -0.57 *P* = 0.13	R = -0.02 *P* = 0.95
Carnitine C13:1	R = 0.09 *P* = 0.82	R = -0.23 *P* = 0.57	R = -0.14 *P* = 0.73	R = -0.19 *P* = 0.65	R = 0.04 *P* = 0.91	R = -0.02 *P* = 0.95	R = -0.14 *P* = 0.72	R = -0.09 *P* = 0.82	R = -0.26 *P* = 0.53	R = -0.19 *P* = 0.64
Carnitine C14:2 Isomer 1	R = -0.07 *P* = 0.86	R = -0.35 *P* = 0.38	R = -0.41 *P* = 0.30	R = -0.23 *P* = 0.57	R = 0.04 *P* = 0.91	R = -0.30 *P* = 0.45	R = -0.26 *P* = 0.52	R = -0.45 *P* = 0.26	R = -0.57 *P* = 0.13	R = -0.02 *P* = 0.95
Guanosine	R = -0.19 *P* = 0.65	R = -0.38 *P* = 0.35	R = -0.41 *P* = 0.30	R = -0.19 *P* = 0.65	R = -0.02 *P* = 0.95	R = -0.50 *P* = 0.20	R = -0.12 *P* = 0.77	R = -0.52 *P* = 0.18	R = -0.50 *P* = 0.20	R = 0.31 *P* = 0.44
Carnitine C6:0 Isomer 1	R = -0.30 *P* = 0.45	R = -0.40 *P* = 0.31	R = -0.49 *P* = 0.21	R = -0.28 *P* = 0.49	R = 0.14 *P* = 0.73	R = -0.59 *P* = 0.11	R = -0.26 *P* = 0.52	R = -0.59 *P* = 0.11	R = -0.71 *P* = 0.04	R = -0.02 *P* = 0.95
Carnitine C6:0	R = -0.28 *P* = 0.49	R = -0.33 *P* = 0.41	R = -0.46 *P* = 0.24	R = -0.21 *P* = 0.61	R = 0.16 *P* = 0.69	R = -0.54 *P* = 0.16	R = -0.24 *P* = 0.56	R = -0.57 *P* = 0.13	R = -0.69 *P* = 0.06	R = -0.09 *P* = 0.81
3-Hydroxykynurenine	R = -0.04 *P* = 0.91	R = 0 *P* = 1	R = -0.07 *P* = 0.86	R = 0.04 *P* = 0.91	R = 0.33 *P* = 0.41	R = -0.28 *P* = 0.49	R = -0.12 *P* = 0.77	R = -0.14 *P* = 0.73	R = -0.11 *P* = 0.77	R = 0.65 *P* = 0.07
3-Hydroxybutyrate	R = -0.35 *P* = 0.38	R = -0.30 *P* = 0.45	R = -0.40 *P* = 0.31	R = -0.42 *P* = 0.28	R = 0.07 *P* = 0.86	R = -0.40 *P* = 0.31	R = -0.39 *P* = 0.33	R = -0.35 *P* = 0.38	R = -0.42 *P* = 0.28	R = 0 *P* = 1
Hexanoyl Glycine	R = 0.09 *P* = 0.82	R = -0.04 *P* = 0.91	R = -0.23 *P* = 0.56	R = -0.02 *P* = 0.95	R = -0.57 *P* = 0.13	R = 0.33 *P* = 0.41	R = -0.31 *P* = 0.44	R = -0.14 *P* = 0.73	R = -0.04 *P* = 0.91	R = -0.60 *P* = 0.10

L-Rhamnose	R = -0.23<br>*P* = 0.57	R = -0.14<br>*P* = 0.73	R = -0.27<br>*P* = 0.50	R = -0.30<br>*P* = 0.45	R = 0.07<br>*P* = 0.86	R = -0.16<br>*P* = 0.69	R = -0.36<br>*P* = 0.37	R = -0.19<br>*P* = 0.65	R = -0.28<br>*P* = 0.49	R = -0.21<br>*P* = 0.60
L-Fucose	R = -0.23<br>*P* = 0.57	R = -0.14<br>*P* = 0.73	R = -0.27<br>*P* = 0.50	R = -0.30<br>*P* = 0.45	R = 0.07<br>*P* = 0.86	R = -0.16<br>*P* = 0.69	R = -0.36<br>*P* = 0.37	R = -0.19<br>*P* = 0.65	R = -0.28<br>*P* = 0.49	R = -0.21<br>*P* = 0.60
1,5-Anhydro-D-Glucitol	R = -0.23<br>*P* = 0.57	R = -0.14<br>*P* = 0.73	R = -0.27<br>*P* = 0.50	R = -0.30<br>*P* = 0.45	R = 0.07<br>*P* = 0.86	R = -0.16<br>*P* = 0.69	R = -0.36<br>*P* = 0.37	R = -0.19<br>*P* = 0.65	R = -0.28<br>*P* = 0.49	R = -0.21<br>*P* = 0.60
Malonicacid	R = -0.19<br>*P* = 0.65	R = -0.19<br>*P* = 0.65	R = -0.25<br>*P* = 0.54	R = -0.28<br>*P* = 0.49	R = 0.11<br>*P* = 0.77	R = -0.23<br>*P* = 0.57	R = -0.24<br>*P* = 0.56	R = -0.19<br>*P* = 0.65	R = -0.26<br>*P* = 0.53	R = 0.04<br>*P* = 0.90
Carnitine C6:0 Isomer 2	R = -0.28<br>*P* = 0.49	R = -0.33<br>*P* = 0.41	R = -0.46<br>*P* = 0.24	R = -0.21<br>*P* = 0.61	R = 0.16<br>*P* = 0.69	R = -0.54<br>*P* = 0.16	R = -0.24<br>*P* = 0.56	R = -0.57<br>*P* = 0.13	R = -0.69<br>*P* =0.06	R = -0.09<br>*P* = 0.81
Hexanoylcarnitine	R = -0.28<br>*P* = 0.49	R = -0.33<br>*P* = 0.41	R = -0.46<br>*P* = 0.24	R = -0.21<br>*P* = 0.61	R = 0.16<br>*P* = 0.69	R = -0.54<br>*P* = 0.16	R = -0.24<br>*P* = 0.56	R = -0.57<br>*P* = 0.13	R = -0.69<br>*P* = 0.06	R = -0.09<br>*P* = 0.81

	Vitamin D3	5,6-dehydroarachidonic acid	B-Pseudouridine	4-Hydroxyphenylpyruvic Acid	Alanine tyrosine	A-Ketoglutaric Acid	Malonicacid	Indole-2-Carboxylic Acid	Tryptamine	3-Hydroxybutyrate
5-Hydroxyindole-3-Acetic Acid	R = -0.40<br>*P* = 0.31	R = -0.27<br>*P* = 0.51	R = -0.26<br>*P* = 0.53	R = -0.05<br>*P* = 0.89	R = 0.09<br>*P* = 0.82	R = -0.30<br>*P* = 0.45	R = -0.80<br>*P* = 0.02	R = -0.10<br>*P* = 0.79	R = -0.07<br>*P* = 0.86	R = -0.69<br>*P* = 0.06
N-Cinnamylglycine	R = -0.48<br>*P* = 0.22	R = -0.43<br>*P* = 0.27	R = -0.40<br>*P* = 0.31	R = -0.32<br>*P* = 0.42	R = -0.29<br>*P* = 0.47	R = -0.42<br>*P* = 0.28	R = -0.47<br>*P* = 0.23	R = -0.30<br>*P* = 0.47	R = -0.42<br>*P* = 0.28	R = -0.26<br>*P* = 0.53
Carnitine C16:1	R = -0.43<br>*P* = 0.27	R = -0.21<br>*P* = 0.60	R = -0.02<br>*P* = 0.95	R = 0.10<br>*P* = 0.79	R = 0.34<br>*P* = 0.40	R = 0.30<br>*P* = 0.45	R = 0<br>*P* = 1	R = 0<br>*P* = 1	R = 0.16<br>*P* = 0.69	R = -0.02<br>*P* = 0.95
Carnitine C15:1	R = -0.20<br>*P* = 0.63	R = 0<br>*P* = 1	R = 0.23<br>*P* = 0.57	R = 0.10<br>*P* = 0.79	R = -0.07<br>*P* = 0.85	R = 0.23<br>*P* = 0.57	R = 0.40<br>*P* = 0.31	R = 0.19<br>*P* = 0.65	R = 0.04<br>*P* = 0.91	R = 0.66<br>*P* = 0.07
Carnitine C14:1	R = -0.01<br>*P* = 0.97	R = 0.10<br>*P* = 0.79	R = 0.30<br>*P* = 0.45	R = 0.21<br>*P* = 0.60	R = 0.23<br>*P* = 0.57	R = 0.45<br>*P* = 0.26	R = 0.30<br>*P* = 0.45	R = 0.19<br>*P* = 0.65	R = 0.26<br>*P* = 0.53	R = 0.35<br>*P* = 0.38
Carnitine C14:2	R = -0.01<br>*P* = 0.97	R = 0.19<br>*P* = 0.65	R = 0.35<br>*P* = 0.38	R = 0.35<br>*P* = 0.38	R = 0.34<br>*P* = 0.40	R = 0.57<br>*P* = 0.13	R = 0.33<br>*P* = 0.41	R = 0.32<br>*P* = 0.42	R = 0.38<br>*P* = 0.35	R = 0.33<br>*P* = 0.41
Carnitine C13:1	R = -0.09<br>*P* = 0.82	R = 0.10<br>*P* = 0.79	R = 0.28<br>*P* = 0.49	R = 0.21<br>*P* = 0.60	R = 0.06<br>*P* = 0.88	R = 0.47<br>*P* = 0.23	R = 0.30<br>*P* = 0.45	R = 0.27<br>*P* = 0.51	R = 0.14<br>*P* = 0.73	R = 0.45<br>*P* = 0.26
Carnitine C14:2 Isomer 1	R = -0.01<br>*P* = 0.97	R = 0.19<br>*P* = 0.65	R = 0.35<br>*P* = 0.38	R = 0.35<br>*P* = 0.38	R = 0.34<br>*P* = 0.40	R = 0.57<br>*P* = 0.13	R = 0.33<br>*P* = 0.41	R = 0.32<br>*P* = 0.42	R = 0.38<br>*P* = 0.35	R = 0.33<br>*P* = 0.41

Guanosine	R = 0.23<br>*P* = 0.57	R = 0.19<br>*P* = 0.65	R = 0.14<br>*P* = 0.73	R = 0.13<br>*P* = 0.74	R = 0.15<br>*P* = 0.71	R = 0.21<br>*P* = 0.61	R = 0.42<br>*P* = 0.28	R = 0.10<br>*P* = 0.79	R = 0.26<br>*P* = 0.53	R = 0.11<br>*P* = 0.77
Carnitine C6:0 Isomer 1	R = 0.20<br>*P* = 0.63	R = 0.24<br>*P* = 0.55	R = 0.30<br>*P* = 0.45	R = 0.30<br>*P* = 0.47	R = 0.40<br>*P* = 0.31	R = 0.52<br>*P* = 0.18	R = 0.28<br>*P* = 0.49	R = 0.21<br>*P* = 0.60	R = 0.33<br>*P* = 0.41	R = 0.23<br>*P* = 0.57
Carnitine C6:0	R = 0.20<br>*P* = 0.63	R = 0.24<br>*P* = 0.55	R = 0.38<br>*P* = 0.35	R = 0.30<br>*P* = 0.47	R = 0.40<br>*P* = 0.31	R = 0.47<br>*P* = 0.23	R = 0.19<br>*P* = 0.65	R = 0.21<br>*P* = 0.60	R = 0.38<br>*P* = 0.35	R = 0.19<br>*P* = 0.65
3-Hydroxykynurenine	R = -0.06<br>*P* = 0.88	R = -0.19<br>*P* = 0.65	R = -0.16<br>*P* = 0.69	R = -0.35<br>*P* = 0.38	R = -0.51<br>*P* = 0.19	R = -0.21<br>*P* = 0.61	R = 0.83<br>*P* = 0.02	R = -0.24<br>*P* = 0.55	R = -0.26<br>*P* = 0.53	R = 0.59<br>*P* = 0.11
3-Hydroxybutyrate	R = 0.21<br>*P* = 0.60	R = 0.24<br>*P* = 0.55	R = 0.28<br>*P* = 0.49	R = 0.08<br>*P* = 0.84	R = -0.29<br>*P* = 0.47	R = 0.26<br>*P* = 0.53	R = 0.88<br>*P* = 0.004	R = 0.24<br>*P* = 0.55	R = -0.02<br>*P* = 0.95	R = 0.97<br>*P* = 3e-05
Hexanoyl Glycine	R = 0.09<br>*P* = 0.82	R = 0.38<br>*P* = 0.35	R = 0.57<br>*P* = 0.13	R = 0.60<br>*P* = 0.11	R = 0.59<br>*P* = 0.12	R = 0.21<br>*P* = 0.61	R = -0.71<br>*P* = 0.04	R = 0.54<br>*P* = 0.16	R = 0.73<br>*P* = 0.03	R = -0.54<br>*P* = 0.16
L-Rhamnose	R = 0.07<br>*P* = 0.85	R = 0.19<br>*P* = 0.65	R = 0.33<br>*P* = 0.41	R = 0.13<br>*P* = 0.74	R = -0.18<br>*P* = 0.65	R = 0.26<br>*P* = 0.53	R = 0.54<br>*P* = 0.16	R = 0.27<br>*P* = 0.51	R = 0.02<br>*P* = 0.95	R = 0.80<br>*P* = 0.02
L-Fucose	R = 0.07<br>*P* = 0.85	R = 0.19<br>*P* = 0.65	R = 0.33<br>*P* = 0.41	R = 0.13<br>*P* = 0.74	R = -0.18<br>*P* = 0.65	R = 0.26<br>*P* = 0.53	R = 0.54<br>*P* = 0.16	R = 0.27<br>*P* = 0.51	R = 0.02<br>*P* = 0.95	R = 0.80<br>*P* = 0.02
1,5-Anhydro-D-Glucitol	R = 0.07<br>*P* = 0.85	R = 0.19<br>*P* = 0.65	R = 0.33<br>*P* = 0.41	R = 0.13<br>*P* = 0.74	R = -0.18<br>*P* = 0.65	R = 0.26<br>*P* = 0.53	R = 0.54<br>*P* = 0.16	R = 0.27<br>*P* = 0.51	R = 0.02<br>*P* = 0.95	R = 0.80<br>*P* = 0.02
Malonicacid	R = 0.10<br>*P* = 0.79	R = 0.13<br>*P* = 0.74	R = 0.23<br>*P* = 0.57	R = -0.02<br>*P* = 0.94	R = -0.43<br>*P* = 0.27	R = 0.14<br>*P* = 0.73	R = 0.85<br>*P* = 0.006	R = 0.16<br>*P* = 0.69	R = -0.09<br>*P* = 0.82	R = 0.95<br>*P* = 2e-04
Carnitine C6:0 Isomer 2	R = 0.20<br>*P* = 0.63	R = 0.24<br>*P* = 0.55	R = 0.38<br>*P* = 0.35	R = 0.30<br>*P* = 0.47	R = 0.40<br>*P* = 0.31	R = 0.47<br>*P* = 0.23	R = 0.19<br>*P* = 0.65	R = 0.21<br>*P* = 0.60	R = 0.38<br>*P* = 0.35	R = 0.19<br>*P* = 0.65
Hexanoylcarnitine	R = 0.20<br>*P* = 0.63	R = 0.24<br>*P* = 0.55	R = 0.38<br>*P* = 0.35	R = 0.30<br>*P* = 0.47	R = 0.40<br>*P* = 0.31	R = 0.47<br>*P* = 0.23	R = 0.19<br>*P* = 0.65	R = 0.21<br>*P* = 0.60	R = 0.38<br>*P* = 0.35	R = 0.19<br>*P* = 0.65

Table S5. Clinical Score Parameters for radiation proctopathy.

Assess the following parameters and tally with associated scoring system:	
A. Physical appearance	
	0 –normal
	1 – lack of grooming
	2 – rough hair coat
	3 – very rough hair coat
B. Posture	
	0 – normal
	1 – sitting in hunched position
	4 – hunched posture, head resting on floor
	6 – lying prone on cage floor/unable to maintain upright posture (**suggests moribund and euthanasia required)
C. Activity/Behavior	
	0 – normal
	1 – somewhat reduced/minor changes in behavior
	3 – above plus change in respiratory rate or effort
D. Hydration	
	0 – normal
	1 – mildly dehydrated (< 1 sec skin tent)
	2 – moderately dehydrated
	(1-2 sec skin tent)
	3 – severely dehydrated
	(> 2 sec skin tent)
E. Body Weight (assessed every two days)	
	0 – normal (<5% change from initial weight)
	1 – 5-10% weight change
	2 – 10-20% weight change
F. Anal hair (determination of the area of hair loss in the anus)	
	0 – normal
	1 – 0 < area < 1
	2 – 1 < area < 2
	3 – 2< area < 4
Endpoint for euthanasia: any single parameter of 6 or combined score for parameters A to F => 15.	
Immediate endpoints for euthanasia:	
	1. Unconsciousness
	2. Inability to remain upright
	3. Agonal respiration (i.e. gasping)
	4. Convulsions

Table S6. The details of correlation between bacteria and fecal metabolites that differentially in WT and RP mice.

Spearman correlation between bacteria and fecal metabolites that differentially in WT and RP mice.

	**Akkermansia muciniphila**	**Lachnospiraceae bacterium_DW59**	**Bacteroides caecimuris**	**Clostridiales Bacterium CIEAF_020**	**Aerococcus urinaeequi**	**Burkholderiales Bacterium YL45**	**Clostridium leptum**	**Clostridium sp Clone.44**	**Proteus vulgaris**	**Mucispirillum schaedleri**	**Klebsiella oxytoca**	**Lactobacillus murinus**	**Enterococcus casseliflavus**	**Citrobacter freundii**
**Carnitine C15:1:DC**	R = -0.72<br>*P* = 1.5e-03	R = -0.58<br>*P* = 0.02	R = -0.65<br>*P* = 0.006	R = -0.46<br>*P* = 0.07	R = -0.41<br>*P* = 0.11	R = -0.62<br>*P* = 0.02	R = -0.40<br>*P* = 0.12	R = -0.50<br>*P* = 0.04	R = 0.45<br>*P* = 0.08	R = 0.64<br>*P* = 0.007	R = 0.41<br>*P* = 0.11	R = 0.30<br>*P* = 0.25	R = 0.31<br>*P* = 0.24	R = 0.39<br>*P* = 0.13
**Carnitine C14:2-OH**	R = -0.68<br>*P* = 3.5e-03	R = -0.46<br>*P* = 0.07	R = -0.45<br>*P* = 0.08	R = -0.50<br>*P* = 0.04	R = -0.20<br>*P* = 0.46	R = -0.51<br>*P* = 0.04	R = -0.45<br>*P* = 0.08	R = -0.57<br>*P* = 0.02	R = 0.53<br>*P* = 0.03	R = 0.73<br>*P* = 0.002	R = 0.43<br>*P* = 0.09	R = 0.10<br>*P* = 0.71	R = 0.27<br>*P* = 0.31	R = 0.40<br>*P* = 0.12
**Carnitine C14:3**	R = -0.60<br>*P* = 1.3e-02	R = -0.43<br>*P* = 0.10	R = -0.44<br>*P* = 0.08	R = -0.41<br>*P* = 0.11	R = -0.08<br>*P* = 0.77	R = -0.35<br>*P* = 0.18	R = -0.46<br>*P* = 0.07	R = -0.38<br>*P* = 0.15	R = 0.36<br>*P* = 0.17	R = 0.51<br>*P* = 0.04	R = 0.30<br>*P* = 0.26	R = 0.13<br>*P* = 0.62	R = 0.21<br>*P* = 0.42	R = 0.26<br>*P* = 0.34
**Carnitine C11:1**	R = -0.70<br>*P* = 2.2e-03	R = -0.61<br>*P* = 0.01	R = -0.57<br>*P* = 0.02	R = -0.52<br>*P* = 0.03	R = -0.13<br>*P* = 0.62	R = -0.47<br>*P* = 0.06	R = -0.51<br>*P* = 0.04	R = -0.60<br>*P* = 0.014	R = 0.58<br>*P* = 0.02	R = 0.65<br>*P* = 0.006	R = 0.56<br>*P* = 0.02	R = 0.21<br>*P* = 0.42	R = 0.43<br>*P* = 0.09	R = 0.53<br>*P* = 0.03
**Prostaglandin E2**	R = -0.55<br>*P* = 2.5e-02	R = -0.35<br>*P* = 0.18	R = -0.41<br>*P* = 0.11	R = -0.60<br>*P* = 0.02	R = -0.24<br>*P* = 0.37	R = -0.55<br>*P* = 0.03	R = -0.39<br>*P* = 0.13	R = -0.40<br>*P* = 0.12	R = 0.43<br>*P* = 0.09	R = 0.58<br>*P* = 0.02	R = 0.42<br>*P* = 0.10	R = -0.06<br>*P* = 0.83	R = 0.14<br>*P* = 0.60	R = 0.34<br>*P* = 0.20
**Carnitine C8:1**	R = -0.54<br>*P* = 2.9e-02	R = -0.47<br>*P* = 0.06	R = -0.46<br>*P* = 0.07	R = -0.35<br>*P* = 0.18	R = -0.03<br>*P* = 0.92	R = -0.31<br>*P* = 0.24	R = -0.38<br>*P* = 0.14	R = -0.48<br>*P* = 0.06	R = 0.33<br>*P* = 0.20	R = 0.55<br>*P* = 0.03	R = 0.30<br>*P* = 0.26	R = 0.29<br>*P* = 0.28	R = 0.35<br>*P* = 0.17	R = 0.30<br>*P* = 0.25
**3-Iodo-L-Tyrosine**	R = -0.55<br>*P* = 2.5e-02	R = -0.40<br>*P* = 0.13	R = -0.44<br>*P* = 0.09	R = -0.52<br>*P* = 0.04	R = -0.07<br>*P* = 0.79	R = -0.33<br>*P* = 0.21	R = -0.46<br>*P* = 0.07	R = -0.26<br>*P* = 0.32	R = 0.39<br>*P* = 0.14	R = 0.51<br>*P* = 0.04	R = 0.35<br>*P* = 0.18	R = 0.04<br>*P* = 0.87	R = 0.13<br>*P* = 0.63	R = 0.33<br>*P* = 0.20
**Decanoyl L-Carnitine**	R = -0.69<br>*P* = 2.8e-03	R = -0.48<br>*P* = 0.06	R = -0.53<br>*P* = 0.03	R = -0.54<br>*P* = 0.03	R = -0.24<br>*P* = 0.38	R = -0.57<br>*P* = 0.02	R = -0.48<br>*P* = 0.06	R = -0.54<br>*P* = 0.03	R = 0.39<br>*P* = 0.13	R = 0.69<br>*P* = 0.003	R = 0.37<br>*P* = 0.16	R = 0.20<br>*P* = 0.46	R = 0.31<br>*P* = 0.24	R = 0.31<br>*P* = 0.24
**Carnitine C12:1**	R = -0.70<br>*P* = 2.4e-03	R = -0.50<br>*P* = 0.04	R = -0.51<br>*P* = 0.04	R = -0.47<br>*P* = 0.07	R = -0.31<br>*P* = 0.24	R = -0.55<br>*P* = 0.03	R = -0.51<br>*P* = 0.04	R = -0.54<br>*P* = 0.03	R = 0.42<br>*P* = 0.10	R = 0.69<br>*P* = 0.003	R = 0.37<br>*P* = 0.15	R = 0.26<br>*P* = 0.33	R = 0.33<br>*P* = 0.21	R = 0.32<br>*P* = 0.22
**Uridine triphosphate**	R = -0.47<br>*P* = 6.3e-02	R = -0.35<br>*P* = 0.17	R = -0.30<br>*P* = 0.26	R = -0.39<br>*P* = 0.13	R = -0.10<br>*P* = 0.72	R = -0.21<br>*P* = 0.42	R = -0.33<br>*P* = 0.21	R = -0.19<br>*P* = 0.48	R = 0.42<br>*P* = 0.10	R = 0.43<br>*P* = 0.09	R = 0.33<br>*P* = 0.20	R = -0.13<br>*P* = 0.63	R = 0.10<br>*P* = 0.72	R = 0.28<br>*P* = 0.28

	Akkermansia muciniphila	Lachnospiraceae bacterium_DW59	Bacteroides caecimuris	Clostridiales Bacterium CIEAF_020	Aerococcus urinaeequi	Burkholderiales Bacterium YL45	Clostridium leptum	Clostridium sp Clone.44	Proteus vulgaris	Mucispirillum schaedleri	Klebsiella oxytoca	Lactobacillus murinus	Enterococcus casseliflavus	Citrobacter freundii
**Vitamin D3**	R = 0.64<br>*P* = 6.9e-03	R = 0.43<br>*P* = 0.09	R = 0.54<br>*P* = 0.03	R = 0.31<br>*P* = 0.24	R = 0.40<br>*P* = 0.13	R = 0.54<br>*P* = 0.03	R = 0.42<br>*P* = 0.11	R = 0.40<br>*P* = 0.13	R = -0.37<br>*P* = 0.16	R = -0.63<br>*P* = 0.009	R = -0.27<br>*P* = 0.31	R = -0.11<br>*P* = 0.69	R = -0.17<br>*P* = 0.51	R = -0.23<br>*P* = 0.39
**5,6-dehydroarachidonic acid**	R = 0.56<br>*P* = 2.3e-02	R = 0.40<br>*P* = 0.12	R = 0.55<br>*P* = 0.03	R = 0.34<br>*P* = 0.19	R = 0.11<br>*P* = 0.67	R = 0.44<br>*P* = 0.08	R = 0.20<br>*P* = 0.45	R = 0.39<br>*P* = 0.13	R = -0.53<br>*P* = 0.04	R = -0.43<br>*P* = 0.09	R = -0.49<br>*P* = 0.06	R = -0.14<br>*P* = 0.61	R = -0.33<br>*P* = 0.21	R = -0.37<br>*P* = 0.15
**B-Pseudouridine**	R = 0.63<br>*P* = 7.9e-03	R = 0.45<br>*P* = 0.08	R = 0.40<br>*P* = 0.12	R = 0.54<br>*P* = 0.03	R = 0.24<br>*P* = 0.37	R = 0.41<br>*P* = 0.11	R = 0.53<br>*P* = 0.03	R = 0.44<br>*P* = 0.08	R = -0.36<br>*P* = 0.16	R = -0.67<br>*P* = 0.004	R = -0.22<br>*P* = 0.41	R = -0.003<br>*P* = 0.99	R = -0.12<br>*P* = 0.67	R = -0.15<br>*P* = 0.56
**4-Hydroxyphenylpyruvic Acid**	R = 0.54<br>*P* = 2.8e-02	R = 0.22<br>*P* = 0.40	R = 0.36<br>*P* = 0.17	R = 0.49<br>*P* = 0.06	R = 0.31<br>*P* = 0.23	R = 0.52<br>*P* = 0.04	R = 0.41<br>*P* = 0.11	R = 0.28<br>*P* = 0.29	R = -0.27<br>*P* = 0.31	R = -0.74<br>*P* = 0.002	R = -0.16<br>*P* = 0.55	R = 0.13<br>*P* = 0.63	R = 0.01<br>*P* = 0.96	R = -0.17<br>*P* = 0.54
**Alanine tyrosine**	R = 0.49<br>*P* = 4.9e-02	R = 0.52<br>*P* = 0.04	R = 0.50<br>*P* = 0.04	R = 0.58<br>*P* = 0.02	R = 0.24<br>*P* = 0.37	R = 0.38<br>*P* = 0.14	R = 0.55<br>*P* = 0.02	R = 0.49<br>*P* = 0.06	R = -0.38<br>*P* = 0.14	R = -0.70<br>*P* = 0.003	R = -0.31<br>*P* = 0.24	R = -0.20<br>*P* = 0.44	R = -0.22<br>*P* = 0.40	R = -0.27<br>*P* = 0.31
**A-Ketoglutaric Acid**	R = 0.57<br>*P* = 2e-02	R = 0.40<br>*P* = 0.12	R = 0.39<br>*P* = 0.13	R = 0.58<br>*P* = 0.02	R = 0.28<br>*P* = 0.28	R = 0.49<br>*P* = 0.06	R = 0.46<br>*P* = 0.07	R = 0.48<br>*P* = 0.06	R = -0.48<br>*P* = 0.06	R = -0.78<br>*P* = 0.0004	R = -0.40<br>*P* = 0.13	R = -0.14<br>*P* = 0.59	R = -0.21<br>*P* = 0.44	R = -0.38<br>*P* = 0.14
**Malonicacid**	R = 0.90<br>*P* = 1.9e-06	R = 0.68<br>*P* = 0.003	R = 0.60<br>*P* = 0.02	R = 0.53<br>*P* = 0.03	R = 0.34<br>*P* = 0.19	R = 0.72<br>*P* = 0.002	R = 0.51<br>*P* = 0.04	R = 0.80<br>*P* = 0.0002	R = -0.65<br>*P* = 0.006	R = -0.74<br>*P* = 0.0009	R = -0.66<br>*P* = 0.005	R = -0.30<br>*P* = 0.25	R = -0.64<br>*P* = 0.007	R = -0.61<br>*P* = 0.02
**3-Hydroxybutyrate**	R = 0.92<br>*P* = 5.2e-07	R = 0.72<br>*P* = 0.002	R = 0.63<br>*P* = 0.008	R = 0.57<br>*P* = 0.02	R = 0.36<br>*P* = 0.17	R = 0.70<br>*P* = 0.003	R = 0.50<br>*P* = 0.04	R = 0.75<br>*P* = 0.0008	R = -0.68<br>*P* = 0.004	R = -0.76<br>*P* = 0.0006	R = -0.68<br>*P* = 0.003	R = -0.29<br>*P* = 0.27	R = -0.63<br>*P* = 0.008	R = -0.66<br>*P* = 0.005
**Indole-2-Carboxylic Acid**	R = 0.53<br>*P* = 3e-02	R = 0.52<br>*P* = 0.04	R = 0.35<br>*P* = 0.19	R = 0.57<br>*P* = 0.02	R = -0.04<br>*P* = 0.88	R = 0.25<br>*P* = 0.35	R = 0.65<br>*P* = 0.007	R = 0.50<br>*P* = 0.04	R = -0.31<br>*P* = 0.24	R = -0.53<br>*P* = 0.03	R = -0.28<br>*P* = 0.29	R = -0.06<br>*P* = 0.82	R = -0.21<br>*P* = 0.43	R = -0.25<br>*P* = 0.35
**Tryptamine**	R = 0.50<br>*P* = 4.7e-02	R = 0.43<br>*P* = 0.09	R = 0.34<br>*P* = 0.19	R = 0.60<br>*P* = 0.02	R = -0.005<br>*P* = 0.98	R = 0.27<br>*P* = 0.30	R = 0.59<br>*P* = 0.02	R = 0.46<br>*P* = 0.07	R = -0.25<br>*P* = 0.35	R = -0.59<br>*P* = 0.02	R = -0.16<br>*P* = 0.55	R = 0.03<br>*P* = 0.91	R = -0.11<br>*P* = 0.67	R = -0.10<br>*P* = 0.70

Table S7. Information of oncology patients

Patients	Sex	Age	Height/cm	Weight/kg	Disease	Medicine[a]	Radiotherapy treatment
#1	Female	46	164	60	Rectal cancer	folfox + PD-1	PTV-GTV 50Gy/25F, PTV-CTV 45Gy/25F
#2	Female	54	160	72	Rectal cancer	folfox	PTV-GTV 50Gy/25F, PTV-CTV 45Gy/25F
#3	Female	60	159	55	Rectal cancer	folfox	PTV-GTV 50Gy/25F, PTV-CTV 45Gy/25F
#4	Female	34	159	44	Rectal cancer	folfox	PTV-GTV 50Gy/25F, PTV-CTV 45Gy/25F
#5	Female	54	165	57	Rectal cancer	folfox	PTV-GTV 50Gy/25F, PTV-CTV 45Gy/25F
#6	Female	64	165	60	Rectal cancer	folfox + PD-1	PTV-GTV 50Gy/25F, PTV-CTV 45Gy/25F
#7	Female	41	161	60	Rectal cancer	folfox	PTV-GTV 50Gy/25F, PTV-CTV 45Gy/25F
#8	Female	59	152	54	Rectal cancer	folfox	PTV-GTV 50Gy/25F, PTV-CTV 45Gy/25F
#9	Female	69	166	55	Rectal cancer	folfox	PTV-GTV 50Gy/25F, PTV-CTV 45Gy/25F
#10	Female	53	160	46	Rectal cancer	folfox	PTV-GTV 50Gy/25F, PTV-CTV 45Gy/25F
#11	Female	71	160	53	Rectal cancer	folfox	PTV-GTV 50Gy/25F, PTV-CTV 45Gy/25F
#12	Female	57	165	58	Rectal cancer	folfox + PD-1	PTV-GTV 50Gy/25F, PTV-CTV 45Gy/25F
#13	Female	48	168	55	Rectal cancer	folfox	PTV-GTV 50Gy/25F, PTV-CTV 45Gy/25F
#14	Female	49	158	53	Rectal cancer	folfox	PTV-GTV 50Gy/25F, PTV-CTV 45Gy/25F
#15	Female	32	160	51	Rectal cancer	folfox	PTV-GTV 50Gy/25F, PTV-CTV 45Gy/25F
#16	Female	79	162	58	Rectal cancer	folfox	PTV-GTV 50Gy/25F, PTV-CTV 45Gy/25F
#17	Female	55	156	50	Rectal cancer	folfox	PTV-GTV 50Gy/25F, PTV-CTV 45Gy/25F
#18	Female	53	158	51	Rectal cancer	folfox	PTV-GTV 50Gy/25F, PTV-CTV 45Gy/25F
#19	Female	64	155	49	Rectal cancer	folfox + PD-1	PTV-GTV 50Gy/25F, PTV-CTV 45Gy/25F
#20	Female	70	166	54	Rectal cancer	folfox	PTV-GTV 50Gy/25F, PTV-CTV 45Gy/25F

a. folfox: Oxaliplatin, Calcium levovorin and Fluorouracil.

Table S8. The primers used in this study.

V3-V4	forward primer 5′-ACTCCTACGGGAGGCAGCA-3′
	reverse primer 5′-GGACTACHVGGGTWTCTAAT-3′
A. muciniphila	forward primer 5′-CAGCACGTGAAGGTGGGGAC-3′
	reverse primer 5′-CCTTGCGGTTGGCTTCAGAT-3′
16S rDNA	forward primer 5′-CGGTGAATACGTTCCCGG-3′
	reverse primer 5′-TACGGCTACCTTGTTACGACTT-3′
q-mGPR43	forward primer 5′-TTGAGCAAGCGGTGGTGAAG-3′
	reverse primer 5′-GGGAGCCCAGTAAGAAAGATGAG-3′
q-mGPR41	forward primer 5′-GCAGCAGAGTGCCAGTTGTCC-3′
	reverse primer 5′-CTTGCCCACGAAGACCACC-3′
q-mGPR40	forward primer 5′-TCTCCTTCGCTCTCTATGTATCTGC-3′
	reverse primer 5′-GAGTCGCAGTTTAGCGTGGGA-3′
q-mGPR109A	forward primer 5′-GTTCGGACTCCTGGGCAATG-3′
	reverse primer 5′-GTCAGGAACGGCAGGCAGAT-3′
q-mGPR81	forward primer 5′-CGCAGAGCGTGAGGGAAAA-3′
	reverse primer 5′-CGTCCCCTACAGAGTTGAAGCCT-3′
q-mGPR35	forward primer 5′-GCACAGTCGCTCCACTTACAGG-3′
	reverse primer 5′-GACCCCAGTCCAGCCTCATTC-3′
q-mIL6	forward primer 5′-GGAGCCCACCAAGAACGATAG-3′
	reverse primer 5′-CCAGCATCAGTCCCAAGAAGG-3′
q-mGAPDH	forward primer 5′-GAGAGTGTTTCCTCGTCCCGTAG-3′
	reverse primer 5′-CAACAATCTCCACTTTGCCACTG-3′
q-hGPR43	forward primer 5′-CCCTCACGAGTTTTGGCTTCTAC-3′
	reverse primer 5′-GCAGTGACCAAAGGACATAACCC-3′
q-hIL6	forward primer 5-′TGTGTGAAAGCAGCAAAGAGGC-3′
	reverse primer 5′-GATGATTTTCACCAGGCAAGTCTC-3′
q-hGAPDH	forward primer 5′-GCGGGGCTCTCCAGAACATC-3′
	reverse primer 5′-GCAGTGGGGACACGGAAGG-3′
p-FabG	forward primer 5′-ATGCAAAAGTTAGCAGGTAA-3′
	reverse primer 5′-CTACATCGTCATGCCTCCGT-3′
q-FabG mRNA	forward primer 5′-ATGAAAGAGGAAGACTGGGATGC-3′
	reverse primer 5′-CGATGTTGCCGACGAGACC-3′
sgRNAs targeting *gpr43*	sgRNA1 5′-AGGCTGCGTCGAACTTCCGC-3′
	sgRNA2 5′-GCTGCCCTTCAAGATCATCG-3′
